# METTL16-mediated inhibition of MXD4 promotes leukemia through activation of the MYC-MAX axis

**DOI:** 10.1038/s41388-025-03563-1

**Published:** 2025-09-14

**Authors:** Guglielmo Bove, Mehrad Babaei, Alberto Bueno-Costa, Sajid Amin, Nicla Simonelli, Rosaria Benedetti, Carmela Dell’Aversana, Mariarosaria Conte, Liliana Montella, Vincenzo Summa, Margherita Brindisi, Maria Rosaria Del Sorbo, Marco Crepaldi, Gregorio Favale, Nuria Profitos-Peleja, Vincenzo Carafa, Gaël Roué, Fortunato Ciardiello, Annalisa Capuano, Hendrik G. Stunnenberg, Wouter L. Megchelenbrink, Angela Nebbioso, Manel Esteller, Lucia Altucci, Nunzio Del Gaudio

**Affiliations:** 1 Department of Precision Medicine, University of Campania “Luigi Vanvitelli”, Vico L. de Crecchio 7, Naples, Italy; 2 Cancer Epigenetics Group, Josep Carreras Leukaemia Research Institute (IJC), IJC Building, Germans Trias I Pujol, Ctra de Can Ruti, Cami de Les Escoles S/N, 08916 Badalona, Barcelona, Catalonia Spain; 3 Program of Medical Epigenetics, Vanvitelli Hospital, Naples, Italy; 4 IEOS-CNR Institute for endocrinology and oncology “Gaetano Salvatore”, Via Sergio Pansini 5, Naples, Italy; 5 Department of Medicine and Surgery, LUM University, Casamassima (BA), Bari, Italy; 6 Medical Oncology Complex Unit, “Santa Maria delle Grazie” Hospital, ASL Napoli 2 Nord, Naples, Italy; 7 Department of Pharmacy, School of Medicine and Surgery, University of Naples “Federico II”, Via D. Montesano 49, Naples, Italy; 8 Lymphoma Translational Group, Josep Carreras Leukaemia Research Institute (IJC), Badalona, Spain; 9 BIOGEM, Via Camporeale Area P.I.P., Ariano Irpino (AV), Italy; 10 Departemnt of Sperimental Medicine, University of Campania “Luigi Vanvitelli”, Vico L. de Crecchio 7, Naples, Italy; 11 Prinses Máxima Centrum, Heidelberglaan 25, Utrecht, The Netherlands; 12 Radboud University, Faculty of Science, Department of Molecular Biology, Nijmegen, The Netherlands; 13 Centro de Investigación Biomédica en Red Cancer (CIBERONC), Madrid, Spain; 14 Instituciò Catalana de Recerca i Estudis Avançats (ICREA), Barcelona, Catalonia Spain; 15 Physiological Sciences Department, School of Medicine and Health Sciences, University of Barcelona (UB), Barcelona, Catalonia Spain; 16 Department of Life Sciences, Health and Health Professions, Link Campus University, Via del Casale Di San Pio V 44, Rome, Italy

**Keywords:** Oncogenes, Cancer genetics

## Abstract

N6-methyladenosine (m^6^A) is an RNA modification that governs multiple aspects of RNA metabolism, including splicing, translation, stability, decay, and the processing of marked transcripts. Although accumulating evidence suggests that the m^6^A writer METTL16 is involved in leukemia, the molecular pathway(s) by which it contributes to leukemogenesis remain unexplored. In this study, we shed light on a novel molecular mechanism whereby METTL16 plays a role in acute myeloid leukemia (AML) progression through an m^6^A-dependent manner. Our investigations revealed that METTL16 is overexpressed in primary AML cells. Genetic depletion of METTL16 or its pharmacological inhibition strongly affected the proliferation of AML cells, eventually triggering apoptosis. Transcriptome-wide analysis identified mRNA of MAX Dimerization Protein 4 (MXD4), a MYC pathway regulator, as a downstream target of METTL16. Mechanistically, we showed that METTL16 controls the stability of MXD4 mRNA, resulting in a reduction in MXD4 protein levels that indirectly activates the MYC-MAX axis, essential for leukemogenesis. Strikingly, the suppression of MXD4 rescued the expression levels of MYC target genes, restoring AML cell survival. Our findings unveil a novel METTL16-MXD4 oncogenic axis crucial for AML progression, establishing small-molecule inhibition of METTL16 as a potential therapeutic approach in leukemia and providing a new strategy to target MYC activity in cancer.

**Figure:**
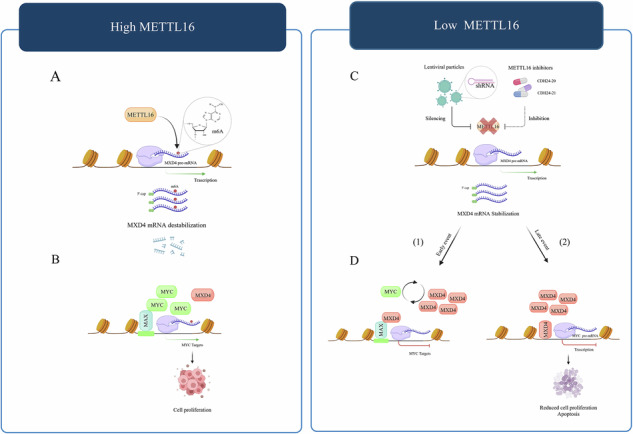
**Molecular model of METTL16-MXD4 axis controlling AML progression by regulating MYC activity. (A)** METTL16 installs m^6^A on MXD4 mRNA, decreasing its stability and resulting in decreased MXD4 protein levels**. (B)** MXD4 reduction ensures MYC-MAX complex formation, MYC target gene expression, and AML cell growth. **(C)** Silencing or chemical inhibition of METTL16 stabilizes MXD4 mRNA and increases its protein levels. **(D)** (*1*) Increased MXD4 proteins may counteract MYC binding with its partner MAX, thus repressing expression of MYC target genes (early event); (*2*) MXD4 binds to MYC regulatory regions, decreasing MYC expression (late event) and affecting AML proliferation.

**Molecular model of METTL16-MXD4 axis controlling AML progression by regulating MYC activity. (A)** METTL16 installs m^6^A on MXD4 mRNA, decreasing its stability and resulting in decreased MXD4 protein levels**. (B)** MXD4 reduction ensures MYC-MAX complex formation, MYC target gene expression, and AML cell growth. **(C)** Silencing or chemical inhibition of METTL16 stabilizes MXD4 mRNA and increases its protein levels. **(D)** (*1*) Increased MXD4 proteins may counteract MYC binding with its partner MAX, thus repressing expression of MYC target genes (early event); (*2*) MXD4 binds to MYC regulatory regions, decreasing MYC expression (late event) and affecting AML proliferation.

## Introduction

Acute Myeloid leukemia is one of the deadliest hematological malignancies, and less than 70% of the patients survive more than 5 years due to drug resistance and relapse [1]. The identification of novel molecular pathways and druggable targets involved in leukemogenesis is therefore essential for developing new anticancer therapeutic strategies. AML is characterized by the disruption of normal hematopoietic differentiation, leading to the selection of neoplastic clones that are unable to differentiate into committed cells [2]. Crosstalk between genomic and epigenomic aberrations generates hematopoietic cell transformation [3], although leukemic cells also acquire epitranscriptomic alterations supporting their uncontrolled proliferation [4]. N^6^-methyladenosine (m^6^A) is the most abundant and well-studied RNA modification [5]. m^6^A influences various aspects of the mRNA life cycle, including RNA stability, degradation, enhancement of translation, and alternative splicing, ultimately affecting gene expression [6]. The turnover of m^6^A is dynamically regulated by two groups of proteins: methyltransferases (*writers*), which place the methylation mark on target RNAs, and demethylases (*erasers*), which remove the modification [7]. Additionally, m^6^A-*reader* proteins, such as members of the YTH domain-containing protein family, bind to m^6^A-modified mRNAs, determining their specific fate [7]. Although accumulating evidence indicates that dysregulation of m^6^A modification and its effectors play a role in the tumorigenesis of several cancer types, including AML [8–10], very little is known about the underlying molecular mechanism by which m^6^A machinery is involved in cancer. METTL16 was recently identified as a novel independent m^6^A writer [11]. METTL16 catalyzes the addition of m^6^A to noncoding RNAs and mRNA transcripts [12, 13]. Recently, METTL16 was associated with AML cell survival via the reprogramming of branched-chain amino acid (BCAA) metabolism [14]. However, the potential impact of METTL16 in supporting the activity of crucial oncogenic pathways required for leukemogenesis is still unexplored. Here, we reveal a novel molecular mechanism by which METTL16 promotes leukemia progression in an m^6^A-dependent manner. We found that METTL16 is overexpressed in primary AMLs. Interfering with METTL16 expression and pharmacologically inhibiting METTL16 both prompted a block in AML progression accompanied by the induction of apoptosis pathways. Mechanistically, we show that METTL16 supports AML proliferation by promoting the instability of MXD4 mRNA, resulting in a decrease in MXD4 protein levels that indirectly activates the MYC-MAX axis, supporting leukemic cell growth. Thus, our findings provide a novel rationale to target MYC activity, opening up new therapeutic perspectives to treat leukemia.

## Materials and methods

### Cell culture and ex vivo cells

K562, U937, NB4, HL-60 and OCI-AML3 cells were purchased from ATCC and grown in RPMI 1640 (Euroclone) supplemented with 10% fetal bovine serum (FBS) (Euroclone), 2 mM L-glutamine (Euroclone), and antibiotics (100 U/mL penicillin, 100 μg/mL streptomycin, and 250 ng/mL amphotericin-B; all Euroclone) at 37 °C and 5% CO_2_. HEK293FT cells were plated in Dulbecco’s Modified Eagle’s Medium (DMEM) (Euroclone) supplemented with 10% FBS, 100 U/mL penicillin/streptomycin (Euroclone), and 2 mM glutamine (Euroclone). Cells have been authenticated by STR analysis.

Leukemic blast cells were isolated from the peripheral blood or bone marrow of leukemia patients and purified using Ficoll (Sigma-Aldrich). Cells were cultured in RPMI 1640 medium (Euroclone) supplemented with 20% heat-inactivated fetal bovine serum (FBS; Sigma-Aldrich), 1% L-glutamine (Euroclone), 1% penicillin/streptomycin (Euroclone), and 0.1% gentamicin (Euroclone). CD34^+^ cells were obtained from STEMCELL Technologies (Catalog No. 70002).

### shRNA design and cloning

The shRNAs specific for *METTL16* and *MXD4* were designed and cloned into pLKO.1-TRC (constitutive) and pLKO.1-Tet-On (inducible) vectors with the AgeI/EcoRI sites. The sequences targeted are as follows:

(shMETTL16.1 and 2) (S)5’CCGGTTGGTCATGCATATGCTTTATCTCGAGATAAAGCATATGCATGACCAATTTTTG-3’(AS)5’AATTCAAAAATTGGTCATGCATATGCTTTATCTCGAGATAAAGCATATGCATGACCAA-3’(S)5’CCGGCGGGGTTGGTATGAAATTAAACTCGAGTTTAATTTCATACCAACCCCGTTTTTG-3’(AS)5’AATTCAAAAACGGGGTTGGTATGAAATTAAACTCGAGTTTAATTTCATACCAACCCCG-3’.

(shMXD4) (S) 5’ CCGG-GT GTCCTTATGTCATTGTAAT-CTCGAG-ATTACAATGACATAAGGACACTTTTTG 3’. (AS) 5’ AATTCAAAAA-GT GTCCTTATGTCATTGTAAT-CTCGAG-ATTACAATGACATAAGGACAC 3’.

### Lentiviral production and infection

For lentiviral production, 5×10^6 HEK293FT cells were seeded in 10 cm culture dishes the previous day. Subsequently, HEK293FT cells were co-transfected with shRNA-targeting plasmids and lentiviral packaging mix plasmids (psPAX2 and pMD2.G in a 3:2:3 ratio), using jetPRIME reagent (Polyplus, #101000046) according to the manufacturer’s protocol. The culture medium was replaced at 24 h post-transfection, and cells were kept in culture for an additional 72 h. Subsequently, 2 mL of the lentivirus-containing supernatant was used to infect 1×10^6 K562, Kasumi-1, and OCI-AML3 cells. Infected cells were kept in an incubator for 30 min and then centrifuged at 1000 RPM for 90 min at 32 °C. After centrifugation, cells were kept in an incubator overnight. The next day, puromycin selection (1 ug/mL) was started and carried out for four days.

### Western blot analysis

WB analysis was performed as previously described [15]. Briefly, RIPA buffer (0.1% SDS, 1% Triton X-100, 150 mM NaCl, 10 mM Tris-HCl pH 8, 1 mM EDTA pH 8) with 1% protease inhibitor cocktail (Roche) was used to lyse the cells. Resulting lysates were subjected to centrifugation for 20 min at 4 °C and heated at 95 °C for 4 min. A total of 25 µg of protein extract was then loaded on SDS-polyacrylamide gel, and proteins were separated through electrophoresis. The resolved proteins were blotted on the nitrocellulose membrane and incubated overnight with target antibodies. The protein signal was visualized by the ECL (Bio-Rad, #1705061).

### Antibodies

Primary antibodies used: anti-METTL16 (Cell Signaling, #17676), anti-MXD4 (Invitrogen, #PA5-40596), anti-PARP (Abcam, #ab32138), anti-MYC (Santa Cruz Biotechnology, #SC764), anti-MAX (Santa Cruz, #SC8011). Antibodies were used according to the manufacturers’ instructions

### RT-qPCR

RNA extraction was performed using a RNeasy Mini Kit (Qiagen, #74104) according to the manufacturer’s instructions. Subsequently, 1 ug of total RNA was reverse transcribed using a Revert Aid RT Kit (Thermo Fisher Scientific, #1621). The cDNA was mixed with SYBR Green (Applied Biosystems, #4367659), and RT-qPCR was run through a Quant Studio™ 3D Digital PCR instrument. Amplification data were analyzed by the ΔΔCt method.

### Colony formation assay

The clonogenic capacity of K562 SCR and shMETTl16 cells was tested with MethoCult H4535 w/o erythropoietin (MethoCult™ GF + H4435) (Stemcell Technologies, #0445) according to the manufacturer’s instructions. SCR and shMETTL16 cells (1×10^4 cells) were mixed with 1 mL of MethoCult in triplicate and seeded in 6-well plates support dishes (Corning, #**3516**). The number of colonies was evaluated after 14 days. Determination of colony number and size was performed with ImageJ software.

### LDH assay

The release of intracellular LDH was assessed with an LDH Assay Kit-WST (Dojindo Laboratories, #CK12) according to the manufacturer’s instructions. A total of 5×10^3 cells in 100 ul were cultured in 96-well plates and kept in an incubator for the indicated time. A total of 10 μl of lysis buffer (Dojindo Laboratories, #CK12) was added to the positive control wells, and the plate was incubated at 37 °C for 30 min. Subsequently, 100 μl of working solution was added to the sample wells, and the plate was incubated at 37 °C for 30 min. Finally, 50 μl stop solution was added and absorbance values were read using the Infinite 200 microplate reader at 490 nm (Tecan).

### In vitro *METTL16* and *METTL3* RNA methylation test

The methylation activity of METTL16 and METTL13 was assessed using the METTL3/METTL14 Complex Chemiluminescent Assay Kit (BPS Bioscience #79614), following the manufacturer’s instructions. The METTL16 purified protein was purchased from Active Motif (#81785) and used according to the kit’s guidelines.

### RNA-seq

The total RNA of the shSCR and shMETTL16 K562 cell lines was purified using a RNeasy Mini Kit (Qiagen, #74104) according to the manufacturer’s instructions. The resulting RNA samples were used to prepare RNA-seq libraries according to the ILLUMINA TruSeq Stranded mRNA protocol. Lastly, cDNA libraries were paired and sequenced using a NovaSeq 6000 instrument (ILLUMINA).

### RNA-seq data processing

The analysis of bulk RNA-seq data was carried out starting from quality assessment and reads mapping onto the human genome GRCh38.p13 (release 39) running STAR [16]. The average read length was 150 bp, and the libraries yielded an average of ~53 million reads for SCR and ~55 million reads for shMETTL16 samples. Bioconductor DESeq2 (https://bioconductor.org/packages/release/bioc/html/DESeq2.html) package running in an R software environment was exploited to normalize raw counts and subsequently to produce their expression characterization. The processed data are summarized in Supplementary Table 1.

### meRIP-seq

meRIP-seq was performed as previously described with a few modifications [17]. Briefly, total RNA was extracted from doxycycline-treated (1 ug/mL for 72 h) SCR and shMETTL16 inducible cells using a RNeasy Mini Kit (Qiagen, #74104) according to the manufacturer’s instructions. Total RNA samples underwent poly-A selection with a Dynabeads mRNA Purification Kit (Thermo Fisher, #61006), and the resulting mRNA was quantified by Nanodrop (Thermo Scientific). Selected mRNA from SCR and shMETTL16 cells was processed according to [17] and then immunoprecipitated overnight with m^6^A antibody (Synaptic System, #202003) and with respective IgG (Santacruz, #sc2027) controls. Protein-A Dynabeads (Thermo Fisher, #1001D) were used to capture the RNA-antibody complex, and the immunoprecipitated RNA was purified using a RNeasy Mini Kit (Qiagen, #74104). RNA-seq libraries were prepared from eluted mRNAs following the standard ILLUMINA mRNA protocol and sent to Novogene (Cambridge, UK) for paired-end sequencing with the NovaSeq 6000 platform (ILLUMINA).

### meRIP-seq data processing

The meRIP-seq data analysis began with 3′-adapter trimming and data quality assessment steps. After trimming, the average read length was ~134 bp for SCR input and IP samples, and ~136 bp for shMETTL16 input and IP samples. Suitable trimmed reads were then mapped to the human genome (GRCh38.p13, release 39) using the STAR aligner [16]. On average, SCR meRIP-seq libraries yielded ~32.5 million paired-end reads, and shMETTL16 libraries yielded ~37.5 million paired-end reads. Peak calling was performed with m6aViewer [18], filtering out duplicate reads and any alignments with a quality score below 37. Peaks with differential p-value cutoff equal to 0.01, height threshold equal to 40, and log_2_FC > 1 were considered significant hyper/hypomethylated peaks. Integrative Genomic Viewer [19] version 2.17.1 was run to visualize significant peaks in the regions of interest. The processed data are presented in Supplementary Table 2

### Gene set enrichment analysis (GSEA)

To better characterize the phenotypical dynamics underlying differentially expressed genes (DEGs), we performed gene set enrichment analysis (GSEA) against the Hallmark biological states and processes gene sets. We included only genes with a minimum read count of 20 in at least one experimental condition. GSEA was used to filter pathways with a p-value < 0.05 and a false discovery rate (FDR) < 0.05 using GSEA_4.3.2 software as made available by the Broad Institute and the University of California, San Diego (https://www.gsea-msigdb.org/gsea/msigdb/index.jsp).

### Gene ontology (GO) enrichment analysis

The biological function of the statistically significant hypomethylated-upregulated genes was analyzed under p-value < 0.05 as a statistical difference screening condition.

Gene Ontology and GO chord diagrams are produced through the Bioinformatics cloud platform (https://www.bioinformatics.com.cn/, an online platform for data analysis and visualization), with all the protein coding genes as the background set.

### ChIP-qPCR

ChIP was performed as previously described [20]. Briefly, cells were crosslinked with formaldehyde (1% final concentration) for 12 min at room temperature. To counteract the effects of formaldehyde, glycine was added to achieve a final concentration of 125 mM. Next, cells were spun for 5 min at 1200 rpm, and the resulting pellet was washed two times with 1X PBS. Cells were subsequently resuspended in lysis buffer B (10 mM EDTA, 0.5 mM EGTA, 20 mM HEPES, pH 7.7, 0.25% Triton X-100) and C (150 mM NaCl, 1 mM EDTA, 50 mM HEPES pH 7.6, 0.5 mM EGTA) and subjected to alternate incubation on rotation at 4 °C for 10 min. An additional centrifugation was carried out to collect nuclei in buffer D (1 mM EDTA, 20 mM HEPES pH 7.6, 0.05% SDS, 0.5 mM EGTA, and protease inhibitors). The resulting nuclei were subjected to sonication using a Bioruptor Pico (Diagenode). Subsequently, samples were spun for 15 min at 13,500 rpm at 4 °C, and supernatants were collected. To perform immunoprecipitation, the following steps were taken: overnight incubation at 4 °C on a rotating wheel with 5 ug of indicated antibody in incubation buffer 1X (150 mM NaCl, 10 mM Tris pH 8, 1 mM EDTA, 1% Triton X-100, 0.5 mM EGTA, 0.15% SDS, protease inhibitors, and 0.1% BSA). Additionally, 5% of each sample was reserved as input for subsequent PCR analysis. The day after, samples were spun at 1200 rpm for 5 min at 4 °C, and 20 ul of Protein A/G PLUS (Santa Cruz Biotechnology, #sc-2003) was added to the incubation mix. The samples were incubated on a rotating wheel at 4 °C for an additional 2 h. Next, the samples were centrifuged at 1200 rpm for 5 min at 4 °C, and the supernatant was discarded after four washes with 500 μL of each wash buffer at 4 °C for 10 min each. After the final wash, 400 μL of elution buffer was added, and elution was carried out for 30 min at room temperature on a rotating wheel. Then, 400 μL of the sample was supplemented with 125 mM NaCl and subjected to overnight de-crosslinking at 65 °C. The following day, protein degradation was achieved by incubating the sample with 40 ug proteinase K, 0.5 M EDTA, and 1 M Tris pH 6.5 at 45 °C for 1 h. Following extraction of DNA using a MinElute Reaction Cleanup Kit (Qiagen, #28204), the subsequent analysis was conducted by real-time PCR on the obtained samples. The indicated antibodies were used for ChIP analysis. Anti-IgG antibodies were used as a negative control.

### Annexin V/PI staining

Allophycocyanin Annexin V/Propidium Iodide assay (Invitrogen, #A35110) was performed according to the supplier’s instructions. Briefly, shRNA-transduced cells (2×10^5 cells) were harvested in cold PBS. Centrifuged cells were resuspended in 100 ul Annexin binding buffer (10 mM HEPES, 2.5 mM CaCl_2_ pH 7.4, and 140 mM NaCl) and adjusted to the concentration of 1×10^6 cells/mL. Subsequently, 5 μl of the Annexin V Conjugated ab was added, and the samples were incubated at room temperature for 15 min. After incubation, 400 μl of Annexin binding buffer and 1 μl of PI (100 μg/mL) were added, and the samples were immediately analyzed. The results were acquired on a BD FACS CantoII flow cytometer system (BD Biosciences).

### Cell counting kit-8 assay (CCK8)

Cell counting Kit-8 (Elabscience, #E-CK-A362) was used to evaluate cell viability. Briefly, 5 × 10^3 cells were seeded in 96-wells plates and kept in an incubator for the indicated time. A total of 10 ul of CCK8 reagent was added to each well, and the plate was incubated for 4 h at 37 °C. Subsequently, absorbance values were read using a Infinite 200 microplate reader (Tecan) at 450 nm.

### Actinomycin D assay

SCR- and shMETTL16-transduced cells were treated with 4 ug/mL Actinomycin D (Sigma Aldrich, #A1410) for the indicated time and then harvested by centrifugation. RNA was extracted using an RNeasy Mini Kit (Qiagen, #74104) and reverse-transcribed with a RevertAid RT Kit (Thermo Fisher, #K1691). The resulting cDNA was used for RT-qPCR analysis.

### Co-immunoprecipitation

Co-immunoprecipitation (Co-IP) of SCR- and shMETTL16-infected cells was performed in non-denature conditions as previously described [20]. In brief, protein lysates were immunoprecipitated with anti-MAX (Santa Cruz, #SC801) and anti-IgG control (Santa Cruz, #sc2025) antibodies. Immunoprecipitated samples were subjected to Protein-G Dynabeads (Thermo Fisher, #1003D) selection. The resulting eluates were run for WB analysis.

### RNA immunoprecipitation

RIP analysis was performed according to [21]. Briefly, 10^6 cells were resuspended in nuclear isolation buffer (40 mM Tris-HCl pH 7.5, 1.28 M sucrose, 20 mM MgCl2, and 4% Triton X-100) and centrifuged for 15 min at 2500 rpm. Next, the nuclear pellets were resuspended in 1 mL RIP buffer (150 mM KCl, 5 mM EDTA, 25 mM Tris pH 7.4, 0.5 mM DTT, 0.5% NP40, 100 U/mL RNAase inhibitor, and protease inhibitors), split into equal fractions (IgG and IP) and sheared through sonication (5 cycles, 30 s off/30 s off). Nuclear membrane and debris were centrifuged at 13,000 rpm for 10 min. The respective antibodies (5 μg) were added to the supernatants and incubated overnight with rotation at 4 °C. Protein-A/G beads were incubated for 1 h at 4 °C. Pelleted beads were then centrifuged at 2500 rpm for 30 sec and washed three times in RIP buffer. RNA was eluted using a RNeasy Mini Kit (Qiagen, #74104) according to the manufacturer’s instructions. The resulting RNA samples were subjected to previous cDNA synthesis for RT-qPCR reaction.

### CAM Assay

Fertilized white Leghorn chicken eggs were obtained from Granja Santa Isabel (Córdoba, Spain) and kept for 8 days at 37 °C with 55% humidity. After eight days of incubation, the eggshell was drilled on top of the air chamber to create a small opening. Subsequently, 1×10^6 SCR and shMETTL16 K562 cells were resuspended in 25 µL of RPMI complete medium (Thermo Fisher Scientific) and 25 µL of Matrigel (BD Biosciences) and then incubated for 15 min at 37 °C. Next, the cell suspensions were injected into the CAM of each egg. Embryos were euthanized by decapitation after 15 days of development. Tumors were excised and weighed, and splenic tissues were collected. DNA was extracted using a DNeasy Blood and Tissue Kit (Qiagen, #69504) and used for RT-qPCR analysis.

### Statistical Analysis

Statistical significance was assessed using unpaired two-tailed Student’s t-test and one-way ANOVA, with a p value < 0.05 considered statistically significant. Details of the statistical tests used, and replicate numbers are provided in the respective figure legends. Statistical tests can be considered appropriate, according to the assessment of normality and variance of data distribution. Neither randomization nor “blinding” of investigators was used.

### Chemical synthesis

#### General methods

For the synthesis of METTL16 inhibitors, starting materials and solvents were purchased from commercial suppliers and used without further purification. For ^1^H NMR and ^13^C NMR measurements, an accurately weighed amount of analyte (about 5.0–10.0 mg) was dissolved in 600 µL of deuterated chloroform (CDCl_3_) or methanol (CD_3_OD). The mixture was transferred into a 5 mm NMR tube and the spectra were acquired on a Bruker Advance 400 MHz or 700 MHz spectrometer, using the residual signal of the deuterated solvent as internal standard. Splitting patterns are described as singlet (s), doublet (d), triplet (t), quartet (q), and broad (br); the values of chemical shifts (δ) are given in ppm and coupling constants (*J*) in Hertz (Hz). NMR data were processed with MestreNova (version 8.1.1, Mestrelab Research). ESI-MS spectra analysis was carried out on an LTQ-XL mass spectrometer. Spectra were recorded by infusion into the ESI source using MeOH as a solvent. Column chromatography was performed using 70–230 mesh, 60 Å pore diameter silica gel. TLC analysis was conducted using 5×20 aluminum foil-supported thin-layer silica gel chromatography plates (F254 indicator) with a thickness of 0.25 mm, using the UV as detection method (225 nM), and ninhydrin and phosphomolybdic acid as staining agents.

#### CDH24-20

To a solution of 5-nitroindole (3.08 mmol, 500 mg) in dry DMSO (1.5 mL), NaH (60% w/w dispersion in mineral oil; 1.48 mmol, 53.28 mg) was added, and the reaction mixture was left under stirring for 1 h. Then, (bromomethyl)benzene (3.7 mol, 0.440 mL) was added, and the reaction mixture left under stirring for 2 h. After the reaction time had elapsed, saturated aqueous ammonium chloride solution was added and washed with EtOAc three times. The organic layers were collected, dried over Na2SO4, and concentrated under reduced pressure. The reaction crude was purified by liquid chromatography on silica gel (Hexane/EtOAc 3:1), affording 1-benzyl-5-nitroindole with 75% yield.

Iron (11 mmol, 621 mg) and ammonium chloride (22.2 mmol, 1.2 g) were added to a solution of 1-benzyl-5-nitroindole (2.22 mmol, 560 mg) in EtOH/H2O (4:1 v/v). Then, the reaction mixture was heated at reflux (80 °C) for 5 h. After the reaction time had elapsed, the solvent was removed under reduced pressure and the reaction mixture’s pH was adjusted with a saturated aqueous NaHCO_3_ solution and extracted three times with DCM. The organic layers were collected and dried over Na_2_SO_4,_ filtered, and concentrated under reduced pressure. The crude reaction was purified by liquid chromatography on silica gel (DCM/MeOH 100:1 v/v) to afford 1-benzylindol-5-amine with 77.7% yield (384 mg).

To a solution of 1-benzylindol-5-amine (1.73 mmol, 384 mg) in dry DMF, pTsOH (1.73 mmol, 298 mg) and sodium dicyanamide (5.1 mmol, 454 mg) were added, and the reaction mixture was allowed to stir for 5 h at 50 °C. After the reaction time had elapsed, water was added to the reaction mixture and the precipitate was filtered and concentrated under reduced pressure to afford 1-(1-benzylindolin-5-yl)-2-cyanoguanidine, which was used in the next reaction step without further purification.

To a solution of compound *1-(1-benzylindolin-5-yl)-2-cyanoguanidine* (0.73 mmol, 497 mg) in dimethoxyethane, boron trifluoride diethyl etherate (8.65 mmol, 1 mL) was added at 0 °C, and the reaction mixture was then heated to 60 °C and allowed to stir for 12 h. After the time reaction had elapsed, volatile components were evaporated under reduced pressure. Then, a solution of 1 M NaOH was added dropwise to the residue dissolved in MeOH and allowed to stir. The crude material was obtained by filtering the precipitate and was then purified by liquid chromatography on silica gel (DCM/MeOH/TEA 100:0.5:0.02 v/v), affording the desired product CDH24-20. ^1^H NMR (400 MHz, CD_3_OD) δ 7.58–7.40 (m, 2H), 7.18–7.10 (m, 2H), 7.02 (d, *J* = 8.8 Hz, 2H), 6.93–6.88 (m, 2H), 5.36 (s, 2H).

#### CDH24-21

Following the same procedure used for the preparation of 1-benzyl-5-nitroindole, 1-(4-chlorobenzyl)-5-nitroindole was obtained starting from 5-nitroindole (1.23 mmol, 200 mg) and 1-(bromomethyl)-4-chlorobenzene (1.48 mol, 303 mg), affording the desired compound with 80% yield. 1-(4-Chlorobenzyl) indolin-5-amine was obtained following the same synthesis procedure used for the preparation of 1-benzylindol-5-amine, starting from 1-(4-chlorobenzyl)-5-nitroindole (0.35 mmol, 100 mg) to afford the desired product with 58% yield (52 mg).

1-(1-(4-Chlorobenzyl)indol-5-yl)-2-cyanoguanidine was obtained following the same synthesis used for the preparation of 1-(1-benzylindolin-5-yl)-2-cyanoguanidine, starting from 1-(4-chlorobenzyl) indolin-5-amine (0.137 mmol, 45 mg) and affording the desired product with quantitative yield. ^1^H NMR (700 MHz, CDCl_3_) δ 8.01 (s, 3H), 7.53 (s, 2H), 7.27 (dd, *J* = 13.8, 6.8 Hz, 7H), 7.24–7.20 (m, 3H), 7.02 (dd, *J* = 19.3, 8.4 Hz, 8H), 6.58 (d, *J* = 2.9 Hz, 2H), 5.32–5.20 (m, 6H).

CDH24-21 was obtained following the same synthesis procedure used for the synthesis of CDH24-20, starting from 1-(1-(4-Chlorobenzyl) indol-5-yl)-2-cyanoguanidine (0.44 mmol, 150 mg) and affording the desired product with 46% yield. ^1^H NMR (400 MHz, CD_3_OD) δ 7.63–7.56 (m, 1H), 7.43–7.39 (m, 1H), 7.18–7.10 (m, 2H), 7.04 (d, *J* = 8.9 Hz, 2H), 6.92 (dd, *J* = 9.1, 4.6 Hz, 2H), 5.40 (s, 2H).

## Results

### METTL16 is overexpressed in AML and sustains leukemic cell proliferation

**Fig. 1:**
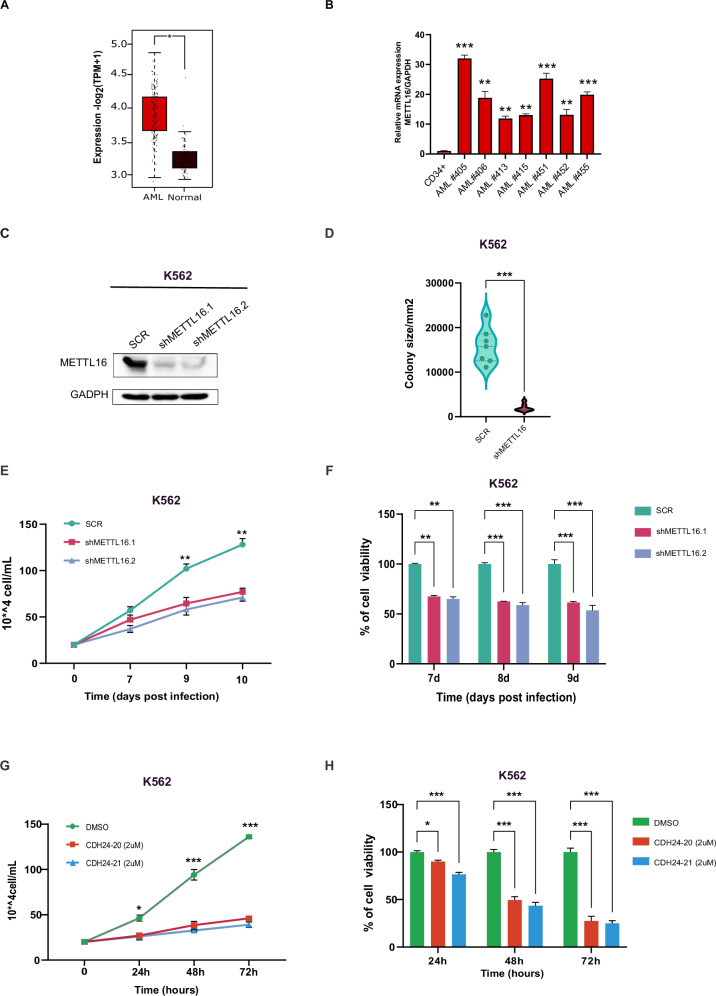
METTL16 is overexpressed in AML, sustaining leukemic cell growth. **A** Expression of METTL16 in AML (LAML) and normal cells from TGCA and GTEx databases (*P < 0.05). **B** RT-qPCR analysis of METTL16 expression in primary AML blasts compared with CD34^+^ (n = 3) (*P^**<**^0.01; ***P < 0.001). **C** Western blot (WB) analysis showing METTL16 protein levels in SCR, shMETTL16.1, and shMETTL16.2 cells upon 4 days of puromycin selection (n = 3). **D** Colony formation assay in SCR and METTL16-depleted K562 cells (n = 3) (***P < 0.001). **E** Proliferation assay of shMETTL16.1-, shMETTL16.2-, and SCR-transduced K562 cells (n = 3) (**P < 0.01). **F** CCK8 viability assay performed in shMETTL16.1-, shMETTL16.2-, and SCR-infected K562 cells (n = 3) (***P < 0.001). **G** Proliferation assay following CDH24-20 and CHD24-21 treatment at the indicated concentrations in K562 cells (n = 3) (***P < 0.001). **H** CCK8 viability assay performed in K562 cells upon treatment with CDH24-20 and CHD24-21, used at the indicated concentrations (n = 3) (***P < 0.001). One-way analysis of variance (ANOVA) non-parametric, was used to calculate the statistical significance of SCR vs shMETTL16.1 and SCR vs METTL16.2 in (**B**, **E**, **F**, **G**, and **H**); Student’s t test was used in (**D**).

To investigate the potential oncogenic role of METTL16 in human cancer, we investigated its expression levels in different clinically annotated cancer types and their non-transformed counterparts (TGCA and GTEx databases [22]; TNMplot analysis). Interestingly, we found that levels of METTL16 were markedly differentially expressed in acute myeloid leukemia (AML) compared to non-transformed myeloid blood cells (Fig. 1A and Supplementary Fig. 1A) [23]. Elevated expression of METTL16 was also observed across various AML subtypes (Supplementary Fig. 1B). High levels of METTL16 in AML were further corroborated by examining METTL16 expression in ex vivo primary human AML blasts compared to CD34^+^ cells. METTL16 was significantly overexpressed in all primary leukemic blasts analyzed, relative to CD34^+^ cells (Fig. 1B and Supplementary Fig. 1C). Upregulation of METTL16 was also observed in leukemic cell lines compared to CD34^+^ (Supplementary Fig. 1D). Given the established role of METTL3 in AML survival [9, 24, 25], we analyzed genome-wide CRISPR-Cas9 knockout (KO) data from different leukemic cell lines in the DepMap database [26]. Noteworthy, we found that the *METTL16* gene is more essential than *METTL3* for AML progression (Supplementary Fig. 1E).

Subsequently, to explore the METTL16 dependency of AML cells we transduced K562, U937, NB4, HL-60 and OCI-AML3 cell lines with lentiviral plasmids harboring two independent short hairpin RNAs (shRNAs) targeting METTL16 (shMETTL16.1 and shMETTL16.2) and a non-targeting shRNA (SCR). We then determined knockdown (KD) efficacy (Fig. 1C, Supplementary Fig. 2A, D, G, L) and carried out phenotypic analyses. Intriguingly, we observed that targeting METTL16 strongly affected rates of AML cell growth compared to SCR (Fig. 1E and Supplementary Fig. 2B, E, H, M). Consistent with AML proliferation arrest, shMETTL16-transduced cells showed a reduced colony formation capability compared to SCR (Fig. 1D and Supplementary Fig. 1F). In addition, depletion of METTL16 was able to impair AML cell viability (Fig. 1F and Supplementary Fig. 2C, F, I, N). Together, these findings indicate that METTL16 is overexpressed in AML and is crucial for leukemic cell growth and proliferation.

Next, we assayed the sensitivity of AML cells to CDH24-20 and CHD24-21, two first-in-class small molecule inhibitors of METTL16 (METTL16i) (patent n. US2022/0339155A1). Both molecules demonstrated high selectivity for METTL16 over METTL3 (Supplementary Fig. 3A) and exhibited a cellular IC50 value of approximately 2 µM (Supplementary Fig. 3B). To further confirm the inhibitors’ specificity, we also analyzed METTL3-regulated gene expression following compound treatment [27]. Remarkably, METTL3 targets were unaffected by CHD24-20 and CHD24-21 (Supplementary Fig. 3C). We next investigated the effects of METTL16-targeting molecules on the phenotype of leukemic cells. Proliferation of K562 cells was markedly reduced upon CDH24-20 and CHD24-21 treatment compared to DMSO-treated (CTRL) cells (Fig. 1G). We also corroborated the growth-inhibitory activity of CDH24-20 and CHD24-21 by evaluating inhibition of cell viability following METTL16i treatment (Fig. 1H).

Taken together, these results further indicate a crucial requirement for METTL16 activity in sustaining AML cell growth that can be efficiently inhibited using CDH24-20 and CHD24-21 molecules.

### Depletion of METTL16 induces programmed cell death in AML

**Fig. 2:**
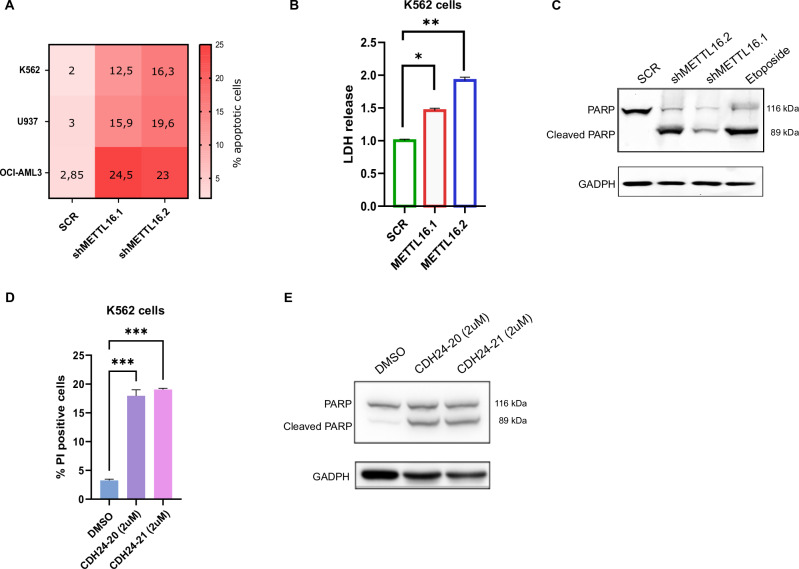
Depletion of METTL16 induces apoptosis in AML. **A** Heatmap showing percentage of Annexin V/PI positive cells in SCR-, shMETTL16.1-, and shMETTL16.2-transduced K562, U937, and OCI-AML3 cell lines (** *P* < 0.01; ***P < 0.001) (n = 3). **B** LDH assay showing cell death in SCR and METTL16-silenced K562 cells (n = 3) (**P < 0.01; *P < 0.05). **C** WB analysis showing *P*ARP protein levels in shMETTL16.1-, shMETTL16.2-, and SCR-transduced K562 cells (n = 3). **D** PI analysis following 72 h treatment with CDH24-20 and CHD24-21 in K562 at the indicated concentrations (n = 3) (***P < 0.001). **E** WB analysis showing PARP protein levels in K452 cells following 72 h treatment with CDH24-20 and CHD24-21 compounds (n = 3). One-way analysis of variance (ANOVA), non-parametric, was used to calculate the statistical significance of SCR vs shMETTL16.1 and SCR vs METTL16.2 in (**A**, **B**, and **D**).

We then examined the induction of cell death in AML cells following METTL16 depletion. We explored apoptosis onset by analyzing the fraction of Annexin V-positive cells. Strikingly, shMETTL16-transduced AML cells showed an increased percentage of apoptotic cells compared to SCR (Fig. 2A and Supplementary Fig. 4A–C). Induction of cell death was also corroborated by the significant increase in lactate dehydrogenase (LDH) release by METTL16-suppressed cells compared to SCR (Fig. 2B). In addition, cleavage of Poly (ADP-ribose) polymerase (PARP) was measured in K562 cells. As expected, METTL16 KD cells revealed the presence of PARP-cleaved fragments (Fig. 2C). Conversely, no activation of PARP was observed in SCR cells (Fig. 2C). Conceivably, CDH24-20 and CHD24-21 treatment triggered apoptosis induction, similarly to the effects observed in shRNA-mediated METTL16 depletion (Fig. 2D, E and Supplementary Fig. 4D). Together, these findings indicate that depletion/inhibition of METTL16 induces apoptosis in AML cells.

### METTL16 regulates crucial pathways involved in leukemic cell survival

**Fig. 3:**
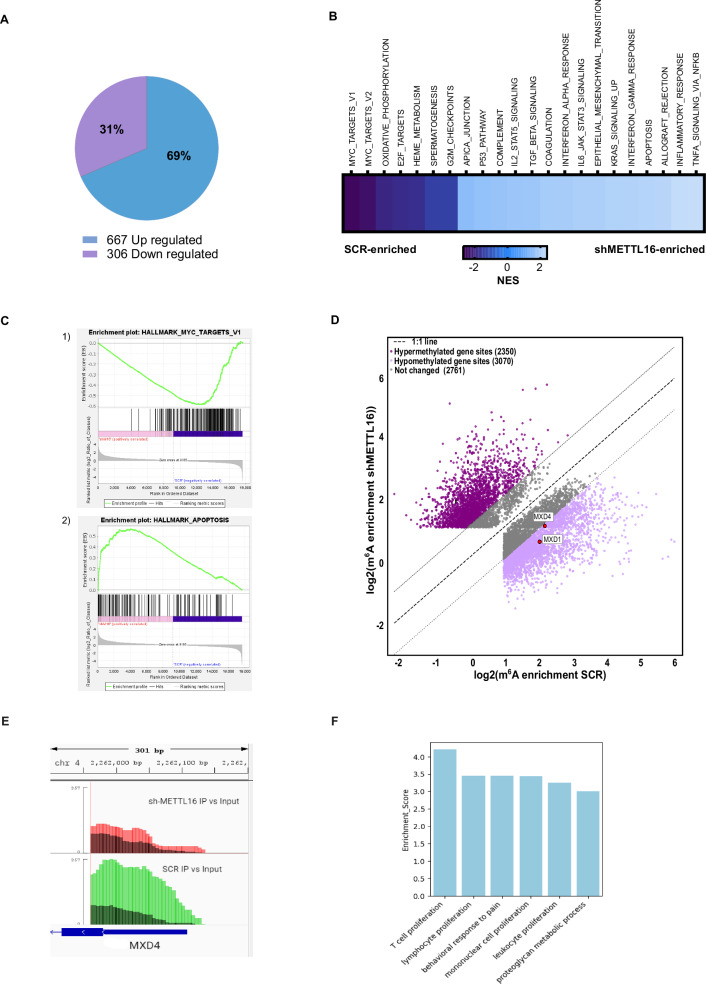
METTL16 silencing affects crucial pathways involved in AML survival and perturbs the m^6^A epitranscriptome of leukemic cells. **A** Pie chart showing the percentage of upregulated and downregulated genes following METTL16 suppression compared to SCR in K562 cells (Log2FC > 1; *P* < 0.05). RNA-seq was carried out after 4 days of puromycin selection. **B** GSEA showing the top differential hallmark gene sets (*P* < 0.05) associated with shMETTL16 and SCR cells (NES indicates the normalized enrichment score). **C** GSEA analysis showing the “MYC targets” and “apoptosis” hallmark gene sets in (1) shMETTL16- (P < 0.01 and FDR < 0.01) and (2) SCR- transduced K562 cells (P < 0.01 and FDR < 0.01). **D** Scatter plot depicting the log2 fold changes methylation gene sites in SCR (x-axis) versus shMETTL16 (y-axis). Methylation in 5′ UTR of MXD4 and exon 6 of MXD1 have been highlighted. **E** Integrative Genomics Viewer (IGV) visualization of m^6^A peak at the 5’UTR region of MXD4 in SCR and shMETTL16 samples (*P* < 0.01). **F** GO analysis of hypomethylated and upregulated genes following METTL16 depletion. Significant enrichment was defined using an unadjusted p value.

To gain insight into the molecular mechanisms by which METTL16 depletion arrests AML cell growth, we conducted RNA sequencing (RNA-seq) experiments on shMETTL16- and SCR-infected K562 cells. A total of 973 deregulated transcripts Abs(Log2FC)>1; p < 0.05 were identified, 306 downregulated (31%) and 667 upregulated (69%) genes following METTL16 depletion (Fig. 3A). Gene Set Enrichment Analysis (GSEA) revealed the top gene sets enriched in SCR and shMETTL16. Interestingly, depletion of METTL16 resulted in suppression of cell proliferation pathways, including MYC and E2F targets, and activation of antiproliferative and pro-apoptotic pathways, such as apoptosis and tumor necrosis factor alpha (TNFα) signaling. (Fig. 3B, C, and Supplementary Fig. 5A, B). Notably, although the MYC target gene set scored as the most affected following METTL16 silencing, MYC expression remained unaltered (Fig. 3B, C and Supplementary Figs. 5B, 7A, 7B).

Together, these findings indicate that METTL16 silencing affects key proliferative pathways in AML.

### Depletion of METTL16 perturbs the m^6^A epitranscriptome in AML

Since METTL16 places m^6^A on target transcripts, we explored the impact of m^6^A dynamics perturbation on gene expression changes driven by shMETTL16. For this purpose, lentiviral vectors harboring Tet-On inducible shMETTL16 plasmids were generated and used to infect K562 cells. SCR- and shMETTL16-transduced cells were then treated with doxycycline (1 μg/mL), and silencing efficiency was evaluated (Supplementary Fig. 6A). Subsequently, to identify on-target mRNA changes in m^6^A, we carried out m^6^A immunoprecipitation followed by high-throughput methylated RNA immunoprecipitation sequencing (meRIP-seq) in shMETTL16- and SCR-transduced K562 cells. Our analysis revealed dynamic variations in m^6^A sites. Overall, the total number of transcripts detected by meRIP-seq with a P value < 0.05 was 7000 for shMETTL16 and 7178 for SCR, respectively. As a result of METTL16 depletion, meRIP-seq analysis revealed that 2350 sites gained m⁶A, whereas 3,070 sites lost the modification (|log₂FC | > 1, P < 0.05; Fig. 3D). Specifically, a total of 1724 genes lost m^6^A (hypomethylated) and 1204 genes gained m^6^A (hypermethylated) following METTL16 depletion (|Log_2_FC ≥ 1 | ; MeRIP‐seq p < 0.01; RNA‐seq adjusted P < 0.05), (Supplementary Fig. 6B). In addition, considering m^6^A distribution, most peaks were equally located at coding sequences (40%) and 3’UTR (40%), while 10% of the peaks were present at 5’UTR, and the remainder were distributed among intronic and intergenic regions (Supplementary Fig. 6C). Interestingly, hypomethylated transcripts were enriched for genes associated with the TNFα signaling pathway (Supplementary Fig. 6D). In addition, GO analysis of concomitantly upregulated and hypomethylated genes (Log_2_FC ≥ 1; p < 0.01) identified a significant enrichment of pathways associated with leukemic proliferation (Fig. 3F and Supplementary Fig. 6B). These results indicate that METTL16 KD perturbs the m6A epitranscriptome, which may in turn influence the expression of selected genes.

### Loss of m^6^A following METTL16 depletion increases RNA stability of MXD4

**Fig. 4:**
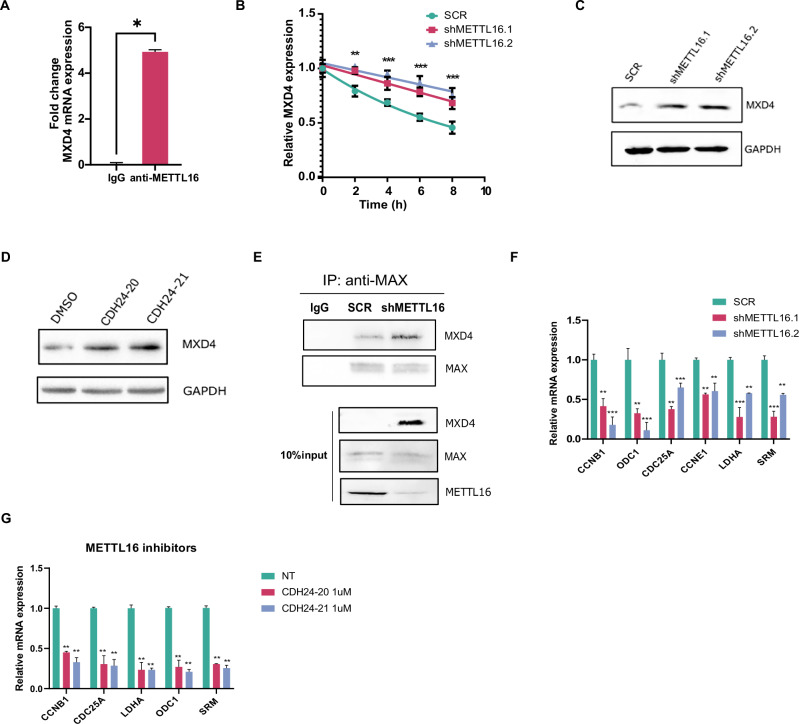
m^6^A loss following METTL16 KD increases RNA stability of MXD4 and suppresses MYC activity. **A** METTL16 RIP assay showing MXD4 RNA enrichment in K562 cells (n = 3) (*P < 0.05). **B** Actinomycin D assay showing MXD4 expression at indicated time points in SCR, shMETTL16.1, and shMETTL16.2 K562 cells (n = 3) (*P < 0,05). **C** WB analysis of MXD4 in SCR-, shMETTL16.1-, and shMETTL16.2-transduced K562 cells (n = 3). **D** WB analysis showing MXD4 protein levels following 48 h treatment with METTL16i (n = 3). **E** IP of MAX in shSCR and shMETTL16 transduced K562 cells at 5 days post-infection (n = 3). Immunoblot was performed with the indicated antibodies. **F** RT-qPCR showing expression of the indicated genes in SCR, shMETTL16.1, and shMETTL16.2 cells at 5 days post -infection (n = 3) (***P < 0.001; **P < 0.01). **G** RT-qPCR showing expression of the indicated genes following 48 h treatment with METTL16i (n = 3) (***P < 0.001; **P < 0.01). One-way analysis of variance (ANOVA), non-parametric, was used to calculate the statistical significance of SCR vs shMETTL16.1 and SCR vs METTL16.2 in (**B**, **F**), and NT vs CDH24-20, NT vs CDH24-21 in (**G**); Student’s t test was used in (**A**).

It is well documented that MYC plays a pivotal role in sustaining the proliferation of leukemic cells. Consequently, identifying novel molecular targets involving MYC regulatory pathways is critical for designing novel anticancer strategies. Given the marked reduction in the expression of MYC target genes without any corresponding change in MYC expression itself (as shown by RNA-seq performed at an early time point; Supplementary Fig. 7A) nor in its methylation status (Supplementary Fig. 7C), we investigated potential alterations in the activity of the MXD family proteins (MXD1, MXD3, MXD4, and MXD5). MXD proteins are known to counteract the oncogenic function of MYC by competing for binding with its transcriptional coactivator MAX and repressing the transcription of MYC target genes [28, 29]. Notably, among the MXD genes that exhibited loss of methylation following METTL16 knockdown, MXD4 emerged as a significantly hypomethylated target (Figs. 3D, E, and Supplementary Fig. 7B). Interestingly, MXD4 showed also a strong inverse correlation with METTL16 (Supplementary Fig. 7D). Therefore, to corroborate the potential binding of METTL16 to MXD4 mRNA, we carried out RNA immunoprecipitation experiments (RIP). Interestingly, the METTL16 pull-down assay followed by Real-time qPCR (RT-qPCR) revealed a strong enrichment of MXD4 mRNA, while no binding was found in the control (Fig. 4A). Since m^6^A loss is frequently associated with alteration of transcript stability causing either stabilization or destabilization [6], an Actinomycin D time course assay was conducted to assess the stability of MXD4 mRNA upon METTL16 KD. Intriguingly, depletion of METTL16 significantly enhanced the stability of MXD4 mRNA (Fig. 4B). In contrast, levels of MXD4 mRNA were sharply reduced in SCR-transduced cells (Fig. 4B). Consequently, levels of MXD4 RNA were found to be upregulated in shMETTL16 compared to the SCR samples (Supplementary Fig. 6E). Next, we analyzed MXD4 protein levels following both METTL16 KD and METTL16 inhibition. As expected, Western Blot (WB) analysis showed a marked time-dependent increase of MXD4 in shMETTL16- compared to SCR-transduced cells (Fig. 4C and Supplementary Fig. 6F). Likewise, an increase in MXD4 was also observed following treatment with CDH24-20 and CHD24-21 in K562 cells (Fig. 4D). Notably, methylation levels of MYC transcript were unaffected upon METTL16 depletion (Supplementary Fig. 7C). Together, these data suggest the involvement of METTL16 in regulating MXD4 expression in an m^6^A-dependent manner.

### METTL16 controls MYC activity via MXD4 mRNA stabilization

**Fig. 5:**
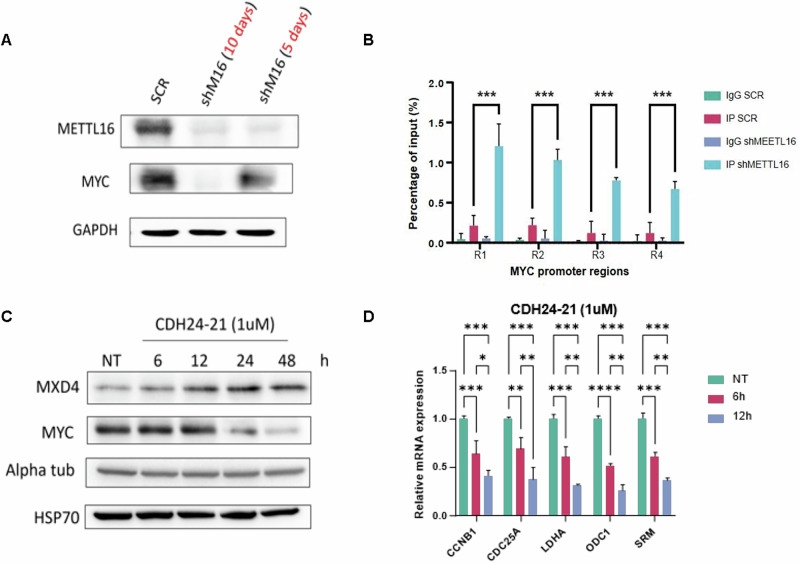
METTL16 depletion impacts on MYC expression. **A** WB analysis of MYC protein levels in SCR- and shMETTL16-transduced cells at 10- and 5-days post-infection, respectively. **B** ChIP-qPCR of MXD4 at MYC-regulated genomic regions (R1-4) in SCR and shMETTL16 cells (n = 3) (***P < 0.001). The experiment was performed after 10 days of puromycin selection. **C** WB analysis showing MXD4 and MYC protein levels following time-points treatment with METTL16i (n = 3). **D** RT-qPCR showing expression of the indicated genes following time-point treatment (6 and 12 h) with METTL16i (n = 3). One-way analysis of variance (ANOVA) non-parametric, was used to calculate the statistical significance of NT vs 6 h and NT vs 12 h in (**D**); Student’s t test was used in (**B**).

We then explored the impact of shMETTL16-driven MXD4 overexpression on MYC activity. We observed a decreased expression of MYC target genes in METTL16-depleted cells compared to the SCR at 5 days post-infection (Fig. 4F, Supplementary Fig. 7B). Similarly, treatment with CDH24-20 and CHD24-21 was able to impair MYC-regulated gene expression (Fig. 4G). Subsequently, we investigated the mechanism underlying MYC inhibition. Immunoprecipitation of MAX showed an increased association of MXD4 to the MYC binding partner MAX, with a concomitant increase in MXD4-MAX complexes in shMETTL16 compared to SCR at 5 days post-infection (Fig. 4E). Surprisingly, we also observed that MXD4 upregulation was associated with reduced MYC protein levels; however, the profile of MYC reduction was found to be time dependent, with disruption of MYC expression occurring only as a late event (10 days post-infection) following METTL16 depletion (Fig. 5A and Supplementary Fig. 6F). Notably, MXD proteins were previously shown to bind the MYC promoter and repress its expression [30]. Consequently, to explore mechanisms underlying MYC silencing (observed as a late event) following METTL16 depletion, we investigated MXD4 occupancy at the MYC promoter region. Intriguingly, chromatin immunoprecipitation-qPCR (ChIP-qPCR) experiments revealed the binding of MXD4 at MYC regulatory sites in shMETTL16-, but not in SCR-transduced cells, at 10 days post-infection (Fig. 5B).

To corroborate the dual (early and late) mechanism(s) of MYC inhibition driven by METTL16 KD, we performed a time-point treatment of leukemic cells with METTL16i (Fig. 5C). Strikingly, treatments at early time points (6 h and 12 h) showed increased protein levels of MXD4, accompanied by no change in MYC expression (Fig. 5C). Therefore, we selected these two time points and analyzed RNA levels of MYC targets. As expected, expression of MYC-driven genes was reduced at 6 h and 12 h post-treatment (Fig. 5D), suggesting the counteracting activity of MXD4 on MYC-MAX complex. On the contrary, late time point treatments (24 h and 48 h) suppressed MYC expression (Fig. 5C), indicating a repression of MYC transcription upon increased MXD4 expression (late event).

**Fig. 6:**
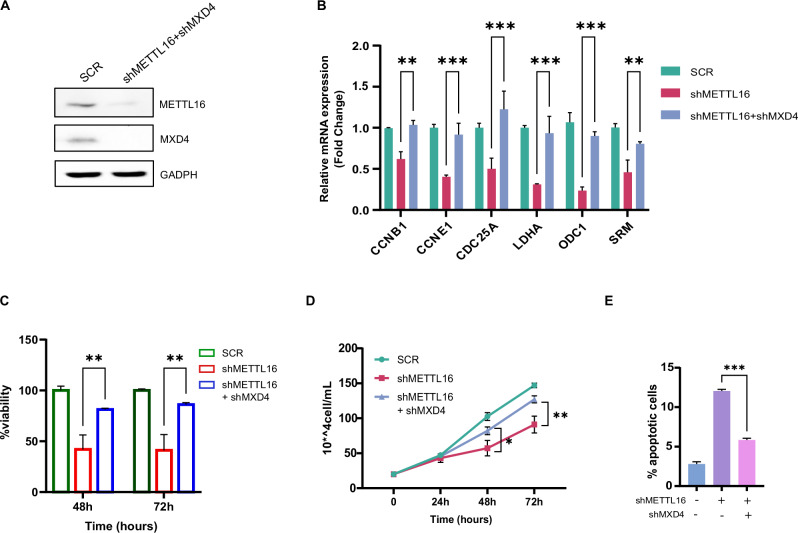
MXD4 depletion rescues MYC activity and AML cell proliferation in shMETTL16 cells. **A** WB analysis of METTL16 and MXD4 protein levels in SCR- and shMETTL16/shMXD4-transduced K562 cells (dual KD). **B** RT-qPCR analysis showing expression of the indicated targets in SCR, METTL16-, and METTL16/MXD4-depleted K562 cells (***P < 0.001; **P < 0.01). (n = 3). **C** CCK8 assay indicating cell viability in SCR-, shMETTL16-, and shMETTL16/shMXD4-transduced K562 cells (**P < 0,01; ***P < 0.001). (n = 3). **D** Proliferation assay performed in SCR-, METTL16-, and METTL16/MXD4-silenced K562 cells (*P < 0,05; **P < 0,01). (n = 3). **E** Annexin V/PI analysis showing apoptosis percentage in SCR-, shMETTL16-, and shMETTL16/shMXD4-infected cells (***P < 0.001) (n = 3). One-way analysis of variance (ANOVA), non-parametric, was used to calculate the statistical significance of shMETTL16 vs shMETTL16+shMXD4 in (**B**, **C**, **D**, and **E**).

To further elucidate the role of MXD4 in the shMETTL16-driven phenotype, we silenced MXD4 expression in shMETTL16-transduced K562 cells (Fig. 6A). Excitingly, suppression of MXD4 was able to rescue the expression of MYC-regulated genes (Fig. 6B). Additionally, dual METTL16 and MXD4 KD restored proliferation rate and cell viability compared to cells infected with shMETTL16 alone (Fig. 6C, D). Interestingly, apoptosis triggered by METTL16 KD was also attenuated upon MXD4 depletion (Fig. 6E and Supplementary Fig. 7E). Notably, shMXD4 alone does not affect the proliferation and apoptosis of leukemic cells (Supplementary Fig. 8A–D). Taken together, these results show that the MXD4-MYC axis crucially promotes leukemic cell arrest following METTL16 depletion.

### Depletion of METTL16 KD impairs leukemogenesis in an *in ovo* xenograft model

**Fig. 7:**
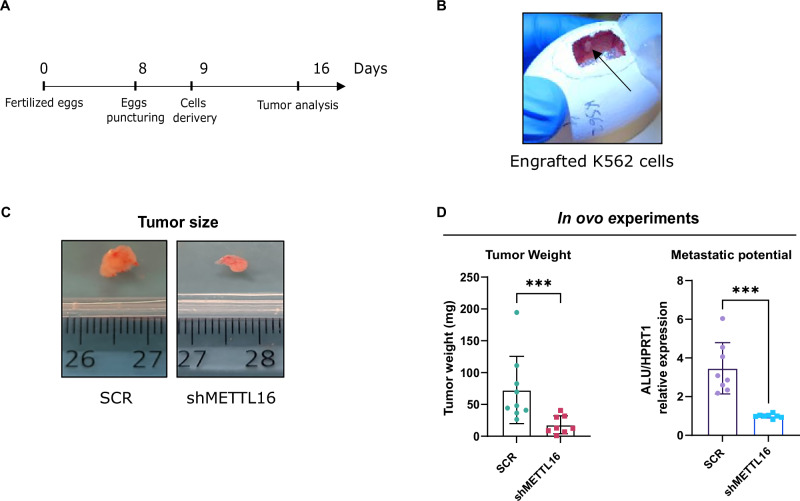
METTL16 KD impairs leukemogenesis in an *in ovo* xenograft model. **A** Timeline and design of the CAM assay experiment. **B** Engraftment of K562 SCR cells on CAM membrane at 9 days of incubation. **C** Visualization of tumor size of SCR- and shMETTL16-excised organoids at 15 days of incubation (***P < 0.001) (n = 8 for SCR and shMETTL16). **D** RT-qPCR showing hALU/ckHPRT1 expression in splenic DNA extracted from SCR- and shMETTL16-inoculated chicken embryos (***P < 0.001). Independent-samples t test was used to calculate statistical significance in (**C**, **D**).

We further investigated the antileukemic efficiency of METTL16 silencing using an *in ovo* xenograft model. This model evaluates the ability of malignant cells to form tumoroids on the top of chicken embryo chorioallantois membrane (CAM) [31]. Initially, SCR and shMETTL16 K562 cells were delivered on the upper CAM membrane, enabling cell engraftment (Fig. 7A, B). Subsequently, the embryos were sacrificed, and both tumor weight and cell metastatic potential were measured (Fig. 7A). As expected, we observed a strong reduction in tumor pellets in shMETTL16- compared to SCR-injected embryos (Fig. 7C). In addition, we quantified engrafted cell distribution along the embryo spleen by RT-qPCR. We specifically analyzed the expression of human- and chicken-related sequences in the spleen [32]. Our results revealed an enrichment of Alu sequences in SCR compared to shMETTL16, supporting a reduced migration of METTL16-depleted cells compared to the control (Fig. 7D). Collectivity, these findings further support the METTL16 dependency of leukemic cells for cancer growth *in ovo*.

## Discussion and conclusion

In recent years, m^6^A has emerged as a crucial regulator of the RNA life cycle and a key determinant of the fate of oncogenic- and oncogenic-related transcripts in tumor context [7]. The main m^6^A writer, METTL3, is reported to be overexpressed in several cancers, including AML [24]. METTL3 was shown to be essential for maintaining the leukemic state and differentiation blockage, but also for supporting AML cell proliferation and conferring tumor chemoresistance [24, 33]. Similarly, the recently identified METTL16, an independent m^6^A methyltransferase, was found to be involved in AML proliferation by enhancing the expression of BCAA aminotransferase genes [14]. However, METTL16 may exert its oncogenic activity and impact on leukemogenesis through multiple molecular axes that are still unexplored. Our study shows that METTL16 is a regulator of the MXD4-MYC-MAX axis, supporting AML tumorigenesis in an m^6^A-dependent manner. By targeting METTL16 we showed its oncogenic role in promoting AML cell growth, which is compatible with the proliferation arrest and apoptosis induction observed in METTL16-depleted cells. Interestingly, unlike METTL3, which is also crucial for leukemic cell survival [24], we found that METTL16 is overexpressed in AML compared to normal cells. However, the mechanisms and pathways driving aberrant METTL16 expression in AML cells were not addressed in this study and will thus require further investigation.

Strikingly, we found that depletion of METTL16 alters the leukemic cell transcriptional program affecting expression of key proliferative genes and enhancing expression of genes involved in apoptosis. Concomitantly, in our experimental setting, meRIP-seq analysis showed that more than 1000 genes lost the m^6^A modification following METTL16 KD, and hundreds of these genes also underwent a significant change in their expression levels (SupplementaryFig. 6B). Although most changes in m⁶A levels did not affect transcript abundance, it is important to consider that m⁶A modifications regulate multiple aspects of RNA metabolism, including processing, nuclear export, splicing, secondary structure modulation, and translation efficiency. Therefore, while many mRNAs undergo methylation, this does not necessarily lead to changes in their steady-state levels. In contrast to previously published article [14], we identified a lower number of hypomethylated transcripts, which might be explained by the potential cell context-dependent activity of METTL16 and/or redundant methylation effects due to METTL3. Notably, both processes are not mutually exclusive.

MYC is an established oncogene that promotes the expression of cell growth-inducing genes enhancing tumor intrinsic and tumor microenvironment interactions, ultimately promoting tumor progression and drug resistance [34]. It also plays a crucial role in the proliferation and progression of AML [35]. Therefore, targeting MYC has the potential to open a clinically therapeutic window for leukemic treatment. However, intrinsic disorders of MYC functional domains and the absence of an enzymatic task have to date precluded structure-driven drug design [36]. Remarkably, the high-affinity interaction between MYC and its transcription partner MAX has contributed to preventing the synthesis of high-potency MYC inhibitors [36]. Here, we describe an alternative manner to target MYC activity via the stabilization of MXD4 mRNA. MXD4 heterodimerizes with MAX and binds the DNA at the same consensus motifs as MYC-MAX, repressing MYC target transcription [37]. Specifically, we demonstrate that silencing or inhibition of METTL16 significantly affects MXD4 mRNA methylation, leading to its stabilization and subsequent increase in MXD4 protein levels. Presumably, the elevated MXD4 protein levels compete with MYC for binding to MAX, thereby counteracting the activity of the MYC-MAX complex, consistent with the corresponding decrease in the activity of MYC. Surprisingly, we also found that METTL16 depletion reduced MYC expression without affecting the methylation of MYC mRNA, suggesting that METTL16 silencing activates other mechanisms that contribute to suppressing MYC activity (Supplementary Fig. 7C). Specifically, we observed that increased levels of MXD4 were associated with reduced MYC expression. Thus, based on previous findings suggesting that MXD proteins can modulate MYC expression [30], we performed ChIP-qPCR analysis and found an increased binding of MXD4 to MYC regulatory regions, indicating the direct involvement of MXD4 in regulating MYC expression. These results suggest a dual layer of MYC regulation upon METTL16 KD. Notably, silencing or inhibition of METTL16 results in a gradual increase in MXD4, which at early time points may compete with MYC for binding MAX, thereby attenuating MYC-MAX activity and down-regulating MYC target genes. While at a later point, MXD4 can directly bind to MYC promoter regions, repressing MYC transcription and contributing to its downregulation. The two experimental approaches, shRNA-mediated silencing and enzymatic targeting of METTL16, differ in their temporal dynamics. In the case of shMETTL16 infection, an increase in MXD4 protein levels is observed at 5 days post-infection (Supplementary Fig. 6F), while in the enzymatic treatment, the increase is observed just 12 h post-treatment (Fig. 5C). At early time points, 5 days post-infection and 12 h post-treatment, we propose that elevated MXD4 competes with MYC for binding MAX (Fig. 4E), leading to the downregulation of MYC target genes without altering MYC mRNA levels. (Fig. 4F, G). Notably, as expected, RNA-seq analysis performed at early time point didn’t show alteration of MYC transcript level (Supplementary Fig. 7A). At later time points, 10 days post-infection and 48 h post-treatment, MXD4 directly binds to the MYC promoter (Fig. 5B), repressing MYC transcription and contributing to the observed downregulation of MYC expression (Fig. 5A, C). Notably, we did not observe a significant reduction in MYC expression following METTL16 depletion in RNA-seq analysis (Supp. Figure 6A), which may depend on the different time frame in which shMETTL16, and SCR cells were harvested and processed. Since MYC is found deregulated in about 70% of human cancers [38] and because of its complex druggability, affecting the MYC pathway by targeting METTL16 represents an attractive opportunity to fight not only leukemia but also, possibly, other cancers whose survival is dependent on MYC expression.

The reversibility of m^6^A offers the possibility to target epitranscriptome alterations, potentially providing new therapeutic approaches. Compounds targeting METTL3 against cancer have been synthesized, and one has already advanced to clinical trials (NCT05584111) [39]. Excitingly, our findings reveal that the first class of METTL16i markedly impairs leukemogenesis, adding further evidence that may speed up the synthesis and optimization of novel molecules inhibiting METTL16 enzymatic activity and possibly allow their future transfer to the clinic. Our study also provides a rationale to indirectly target MYC activity, paving the way to novel therapeutic perspectives in AML.

In conclusion, we show that METTL16 supports AML proliferation by promoting the instability of MXD4 mRNA, which indirectly activates the MYC-MAX complex, crucial for AML cell growth. Our findings indicate possible avenues for the indirect modulation of MYC activity, which may guide future investigation into therapeutic strategies for AML.

## Supplementary information

Supplemetary Figures

Supplementary Table 1

Supplementary Table 2

Primers list

## Data Availability

To ensure full data accessibility, all relevant data have been uploaded to NCBI’s BioProject (ID: PRJNA1103389).

## References

[1] Döhner H, Weisdorf DJ, Bloomfield CD. Acute Myeloid Leukemia. N Engl J Med. 2015;373:1136–52. 26376137 10.1056/NEJMra1406184

[2] Gilliland DG. Targeted therapies in myeloid leukemias. Ann Hematol. 2004;83 Suppl 1:S75–76. 15124682 10.1007/s00277-004-0850-2

[3] Plass C, Oakes C, Blum W, Marcucci G. Epigenetics in acute myeloid leukemia. Semin Oncol. 2008;35:378–87. 18692688 10.1053/j.seminoncol.2008.04.008 PMC3463865

[4] Barbieri I, Kouzarides T. Role of RNA modifications in cancer. Nat Rev Cancer. 2020;20:303–22. 32300195 10.1038/s41568-020-0253-2

[5] Ji P, Wang X, Xie N, Li Y. N6-methyladenosine in RNA and DNA: an epitranscriptomic and epigenetic player implicated in determination of stem cell fate. Stem cells Int. 2018;2018:3256524. 30405719 10.1155/2018/3256524 PMC6199872

[6] Zaccara S, Ries RJ, Jaffrey SR. Reading, writing and erasing mRNA methylation. Nat Rev Mol Cell Biol. 2019;20:608–24. 31520073 10.1038/s41580-019-0168-5

[7] Deng LJ, Deng WQ, Fan SR, Chen MF, Qi M, Lyu WY, et al. m6A modification: recent advances, anticancer targeted drug discovery and beyond. Mol cancer. 2022;21:52. 35164788 10.1186/s12943-022-01510-2 PMC8842557

[8] Hong YG, Yang Z, Chen Y, Liu T, Zheng Y, Zhou C, et al. The RNA m6A reader YTHDF1 is required for acute myeloid leukemia progression. Cancer Res. 2023;83:845–60. 36634204 10.1158/0008-5472.CAN-21-4249

[9] Vu LP, Pickering BF, Cheng Y, Zaccara S, Nguyen D, Minuesa G, et al. The N(6)-methyladenosine (m(6)A)-forming enzyme METTL3 controls myeloid differentiation of normal hematopoietic and leukemia cells. Nat Med. 2017;23:1369–76. 28920958 10.1038/nm.4416 PMC5677536

[10] Weng H, Huang F, Yu Z, Chen Z, Prince E, Kang Y, et al. The m(6)A reader IGF2BP2 regulates glutamine metabolism and represents a therapeutic target in acute myeloid leukemia. Cancer cell. 2022;40:1566–1582.e1510. 36306790 10.1016/j.ccell.2022.10.004 PMC9772162

[11] Brown JA, Kinzig CG, DeGregorio SJ, Steitz JA. Methyltransferase-like protein 16 binds the 3’-terminal triple helix of MALAT1 long noncoding RNA. Proc Natl Acad Sci USA. 2016;113:14013–8. 27872311 10.1073/pnas.1614759113 PMC5150381

[12] Doxtader KA, Wang P, Scarborough AM, Seo D, Conrad NK, Nam Y. Structural basis for regulation of METTL16, an S-adenosylmethionine homeostasis factor. Mol cell. 2018;71:1001–1011.e1004. 30197297 10.1016/j.molcel.2018.07.025 PMC6367934

[13] Su R, Dong L, Li Y, Gao M, He PC, Liu W, et al. METTL16 exerts an m(6)A-independent function to facilitate translation and tumorigenesis. Nat cell Biol. 2022;24:205–16. 35145225 10.1038/s41556-021-00835-2 PMC9070413

[14] Han L, Dong L, Leung K, Zhao Z, Li Y, Gao L, et al. METTL16 drives leukemogenesis and leukemia stem cell self-renewal by reprogramming BCAA metabolism. cell stem cell. 2023;30:52–68.e13. 36608679 10.1016/j.stem.2022.12.006 PMC9838187

[15] Di Costanzo A, Del Gaudio N, Conte L, Dell’Aversana C, Vermeulen M, de Thé H, et al. The HDAC inhibitor SAHA regulates CBX2 stability via a SUMO-triggered ubiquitin-mediated pathway in leukemia. Oncogene. 2018;37:2559–72. 29467492 10.1038/s41388-018-0143-1 PMC5945585

[16] Dobin A, Davis CA, Schlesinger F, Drenkow J, Zaleski C, Jha S, et al. STAR: ultrafast universal RNA-seq aligner. Bioinforma (Oxf, Engl). 2013;29:15–21. 10.1093/bioinformatics/bts635 PMC3530905 23104886

[17] Dominissini D, Moshitch-Moshkovitz S, Salmon-Divon M, Amariglio N, Rechavi G. Transcriptome-wide mapping of N(6)-methyladenosine by m(6)A-seq based on immunocapturing and massively parallel sequencing. Nat Protoc. 2013;8:176–89. 23288318 10.1038/nprot.2012.148

[18] Antanaviciute A, Baquero-Perez B, Watson CM, Harrison SM, Lascelles C, Crinnion L, et al. m6aViewer: software for the detection, analysis, and visualization of N(6)-methyladenosine peaks from m(6)A-seq/ME-RIP sequencing data. RNA (N. Y, NY). 2017;23:1493–501. 10.1261/rna.058206.116 PMC5602108 28724534

[19] Robinson JT, Thorvaldsdóttir H, Winckler W, Guttman M, Lander ES, Getz G, et al. Integrative genomics viewer. Nat Biotechnol. 2011;29:24–26. 21221095 10.1038/nbt.1754 PMC3346182

[20] Del Gaudio N, Di Costanzo A, Liu NQ, Conte L, Migliaccio A, Vermeulen M, et al. BRD9 binds cell type-specific chromatin regions regulating leukemic cell survival via STAT5 inhibition. Cell death Dis. 2019;10:338. 31000698 10.1038/s41419-019-1570-9 PMC6472371

[21] Khalil AM, Guttman M, Huarte M, Garber M, Raj A, Rivea Morales D, et al. Many human large intergenic noncoding RNAs associate with chromatin-modifying complexes and affect gene expression. Proc Natl Acad Sci USA. 2009;106:11667–72. 19571010 10.1073/pnas.0904715106 PMC2704857

[22] Ley TJ, Miller C, Ding L, Raphael BJ, Mungall AJ, Robertson A, et al. Genomic and epigenomic landscapes of adult de novo acute myeloid leukemia. N. Engl J Med. 2013;368:2059–74. 23634996 10.1056/NEJMoa1301689 PMC3767041

[23] Bartha A, Gyorffy B. TNMplot.com: a web tool for the comparison of gene expression in normal, tumor and metastatic tissues. Int J Mol Sci. 2021;22:2622. 33807717 10.3390/ijms22052622 PMC7961455

[24] Barbieri I, Tzelepis K, Pandolfini L, Shi J, Millán-Zambrano G, Robson SC, et al. Promoter-bound METTL3 maintains myeloid leukaemia by m(6)A-dependent translation control. Nature. 2017;552:126–31. 29186125 10.1038/nature24678 PMC6217924

[25] Sang L, Wu X, Yan T, Naren D, Liu X, Zheng X, et al. The m(6)A RNA methyltransferase METTL3/METTL14 promotes leukemogenesis through the mdm2/p53 pathway in acute myeloid leukemia. J Cancer. 2022;13:1019–30. 35154467 10.7150/jca.60381 PMC8824895

[26] Tsherniak A, Vazquez F, Montgomery PG, Weir BA, Kryukov G, Cowley GS, et al. Defining a cancer dependency map. Cell. 2017;170:564–576.e516. 28753430 10.1016/j.cell.2017.06.010 PMC5667678

[27] Ianniello Z, Sorci M, Ceci Ginistrelli L, Iaiza A, Marchioni M, Tito C, et al. New insight into the catalytic -dependent and -independent roles of METTL3 in sustaining aberrant translation in chronic myeloid leukemia. Cell death Dis. 2021;12:870. 34561421 10.1038/s41419-021-04169-7 PMC8463696

[28] Cho S, Lee G, Pickering BF, Jang C, Park JH, He L, et al. mTORC1 promotes cell growth via m(6)A-dependent mRNA degradation. Mol cell. 2021;81:2064–2075.e2068. 33756105 10.1016/j.molcel.2021.03.010 PMC8356906

[29] Mathsyaraja H, Freie B, Cheng PF, Babaeva E, Catchpole JT, Janssens D, et al. Max deletion destabilizes MYC protein and abrogates Eµ-Myc lymphomagenesis. Genes Dev. 2019;33:1252–64. 31395740 10.1101/gad.325878.119 PMC6719623

[30] Lee TC, Ziff EB. Mxi1 is a repressor of the c-Myc promoter and reverses activation by USF. J Biol Chem. 1999;274:595–606. 9872993 10.1074/jbc.274.2.595

[31] Ribatti D. The chick embryo chorioallantoic membrane (CAM) assay. Reprod Toxicol (Elmsford, NY). 2017;70:97–101. 10.1016/j.reprotox.2016.11.004 27832950

[32] Rousset X, Maillet D, Grolleau E, Barthelemy D, Calattini S, Brevet M. Embryonated chicken tumor xenografts derived from circulating tumor cells as a relevant model to study metastatic dissemination: a proof of concept. Cancers. 2022;14:4085 36077622 10.3390/cancers14174085 PMC9454737

[33] Wu X, Ye W, Gong Y. The role of RNA methyltransferase METTL3 in normal and malignant hematopoiesis. Front Oncol. 2022;12:873903. 35574332 10.3389/fonc.2022.873903 PMC9095908

[34] Dhanasekaran R, Deutzmann A, Mahauad-Fernandez WD, Hansen AS, Gouw AM, Felsher DW. The MYC oncogene - the grand orchestrator of cancer growth and immune evasion. Nat Rev Clin Oncol. 2022;19:23–36. 34508258 10.1038/s41571-021-00549-2 PMC9083341

[35] Delgado MD, León J. Myc roles in hematopoiesis and leukemia. Genes Cancer. 2010;1:605–16. 21779460 10.1177/1947601910377495 PMC3092227

[36] Llombart V, Mansour MR. Therapeutic targeting of “undruggable” MYC. EBioMedicine. 2022;75:103756. 34942444 10.1016/j.ebiom.2021.103756 PMC8713111

[37] Diolaiti D, McFerrin L, Carroll PA, Eisenman RN. Functional interactions among members of the MAX and MLX transcriptional network during oncogenesis. Biochimica et biophysica acta. 2015;1849:484–500. 24857747 10.1016/j.bbagrm.2014.05.016 PMC4241192

[38] Dang CV. MYC on the path to cancer. Cell. 2012;149:22–35. 22464321 10.1016/j.cell.2012.03.003 PMC3345192

[39] Yankova E, Blackaby W, Albertella M, Rak J, De Braekeleer E, Tsagkogeorga G, et al. Small-molecule inhibition of METTL3 as a strategy against myeloid leukaemia. Nature. 2021;593:597–601. 33902106 10.1038/s41586-021-03536-w PMC7613134

