# Supplementary material for: METTL16-mediated inhibition of MXD4 promotes leukemia through activation of the MYC-MAX axis
Source: Oncogene. 2025 Sep 14;44(43):4159–72. doi: 10.1038/s41388-025-03563-1 (PMC12537489; doi:10.1038/s41388-025-03563-1)
Supplement: Supplementary file 1 — Supplemetary Figures [file 41388_2025_3563_MOESM1_ESM.pdf]

Supplementary figure 1

A

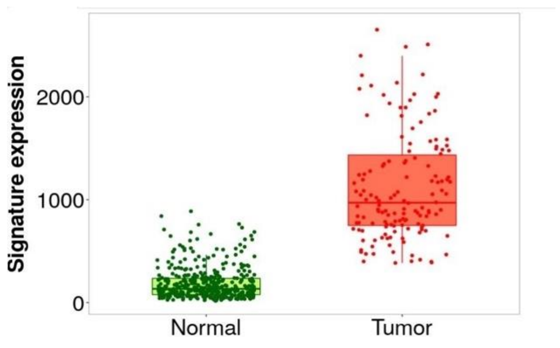

Mann_Whitney_p	FC_T.N	N_genes
1.65e-70	7.59	1

B

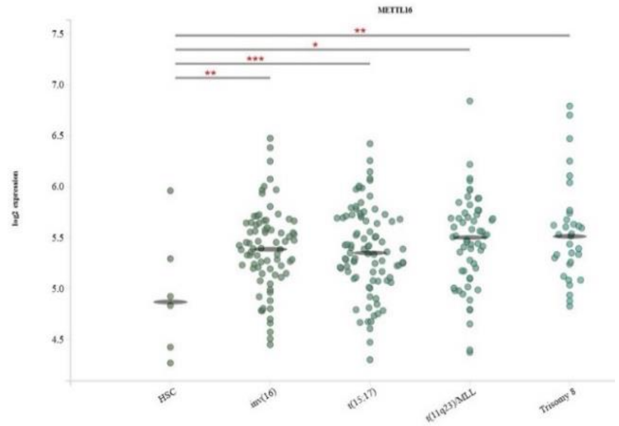

C

Patient ID	Diagnosis	Age	Source
405	AML	62	Bone Marrow
406	AML	87	Bone Marrow
413	AML	56	Peripheral blood
415	AML	71	Peripheral blood
451	ALL (b Ph+)	42	Bone Marrow
452	T-ALL	25	Peripheral blood
455	AML	73	Bone Marrow

D

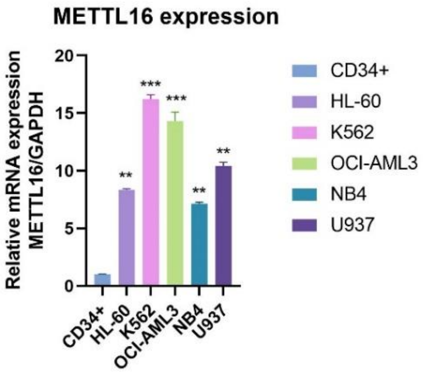

E

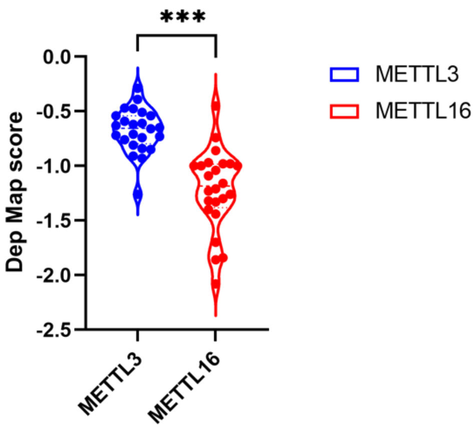

F

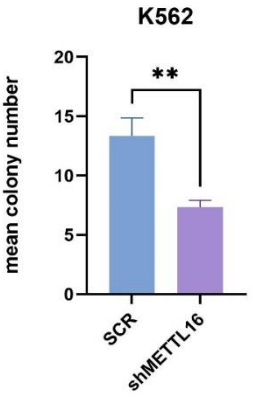

Figure S1. METTL16 expression and dependency in AML. (A) METTL16 expression in normal and tumor AML samples from TNMplot database (T=151, N=407; ***P<0.001). **(B)** Overexpression of METTL16 in AML subtypes from Bloodspot database (*P<0.05;**P<0.01; ***P< 0.001). **(C)** Table summarizing the clinical features, age, diagnosis, and sample source of the seven leukemic patients. **(D)** RT-qPCR analysis of METTL16 expression in leukemic cell lines compared with CD34+ (n=3; **P<0.01; ***P< 0.001). **(E)** AML cell dependency from METTL3 and METTL16 based on DepMap analysis (***P<0.001). **(F)** Mean colony number in SCR and shMETTL16 K562 cells (n=3; **P<0.01). Two-way analysis of variance (ANOVA), non-parametric, was used to calculate the statistical significance of CD34+ vs each AML cell in D; Student's t-test was used in E and F.

Supplementary figure 2

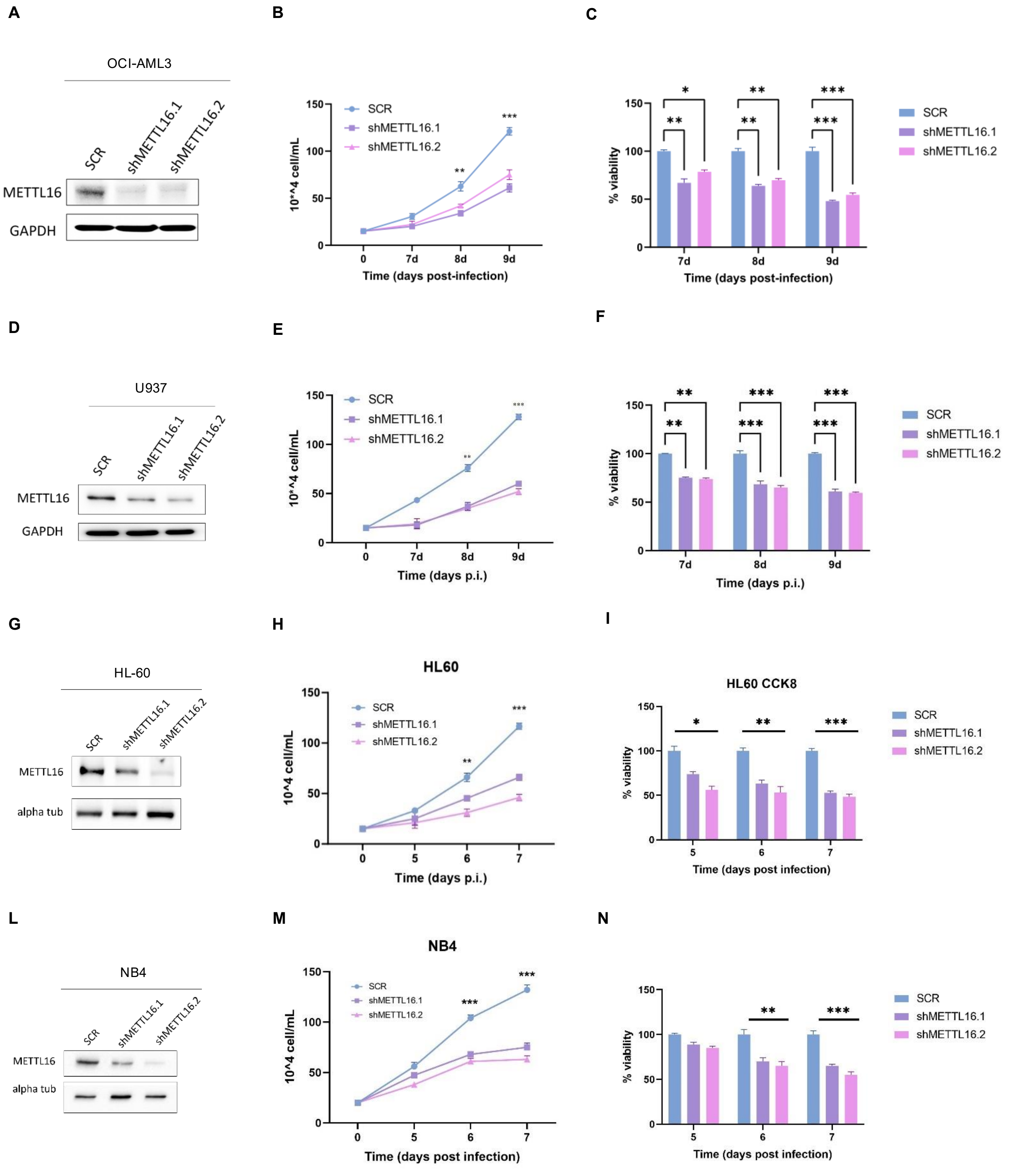

Figure S2. METTL16 depletion affects leukemic cell proliferation. (A, D, G, and L) WB analysis showing METTL16 protein levels in SCR-, shMETTL16.- and shMETTL16.2-transduced OCI.-AML3 (A), U937 (D), HL-60 (G) and NB4 (L) cells. (n=3) **(B, E, H, and M)** Proliferation assay of shMETTL16.1-, shMETTL16.2-, and SCR-transduced OCI-AML3 (B), U937 (E), HL-60 (H) and NB4 (M) cell lines (*p<0.05;**$p \leq 0.01$, ,***P<0.001). (n=3) **(C, F, I and N)** CCK8 viability assay in shMETTL16.1-, shMETTL16.2-, and SCR-infected OCI-AML3 (C), U937 (F), HL-60 (I) and NB4 (N) cell lines (*P<0.05, **$P \leq 0.01$,***P<0.001) (n=3). One-way analysis of variance (ANOVA), non-parametric, was used to calculate the statistical significance of SCR vs shMETTL16.1 and SCR vs METTL16.2 in B, C, E, F, H, I, L, and M.

Supplementary figure 3

A

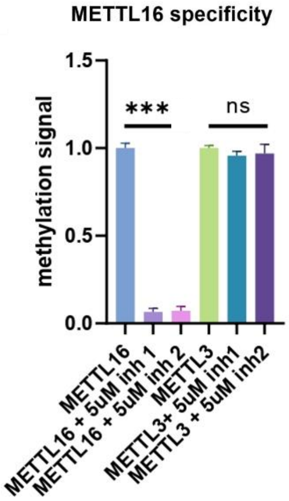

B

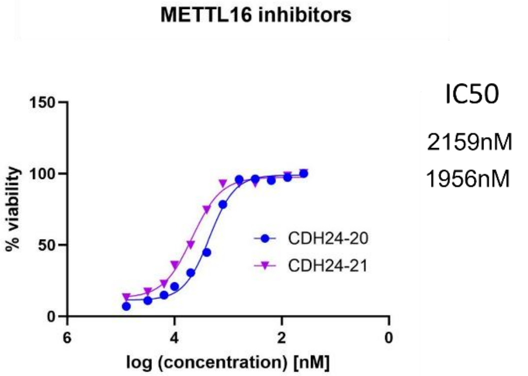

C

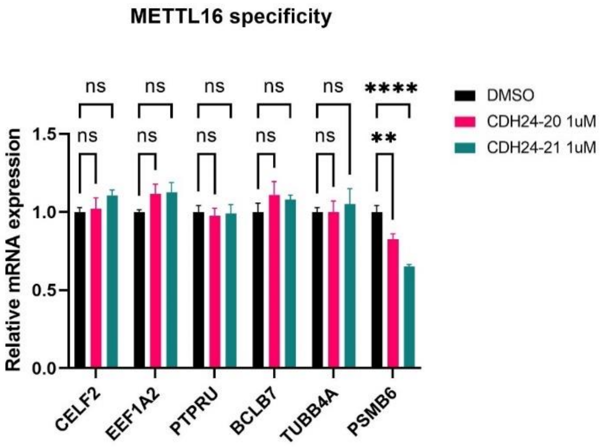

Figure S3. CDH24-20 and -21 specifically target METTL16 (A) RNA methylation assay evaluating *in vitro* methylation activity of METTL16 and METTL3 (***P<0.001) (n=3). **(B)** Dose-response curves of CDH24-20 and CDH24-21 performed in K452 at 48h (n=3). **(C)** RT-qPCR showing the expression of METTL3-regulated genes following 48h of CDH24-20 and-21 treatment (**$P\leq$0.01,***P<0.001) (n=3). One-way analysis of variance (ANOVA) non-parametric, was used to calculate the statistical significance of METTL16 vs METTL16 inh1/2 in A and DMSO vs CDH24-20 and DMSO vs CDH24-21 in C.

Supplementary figure 4

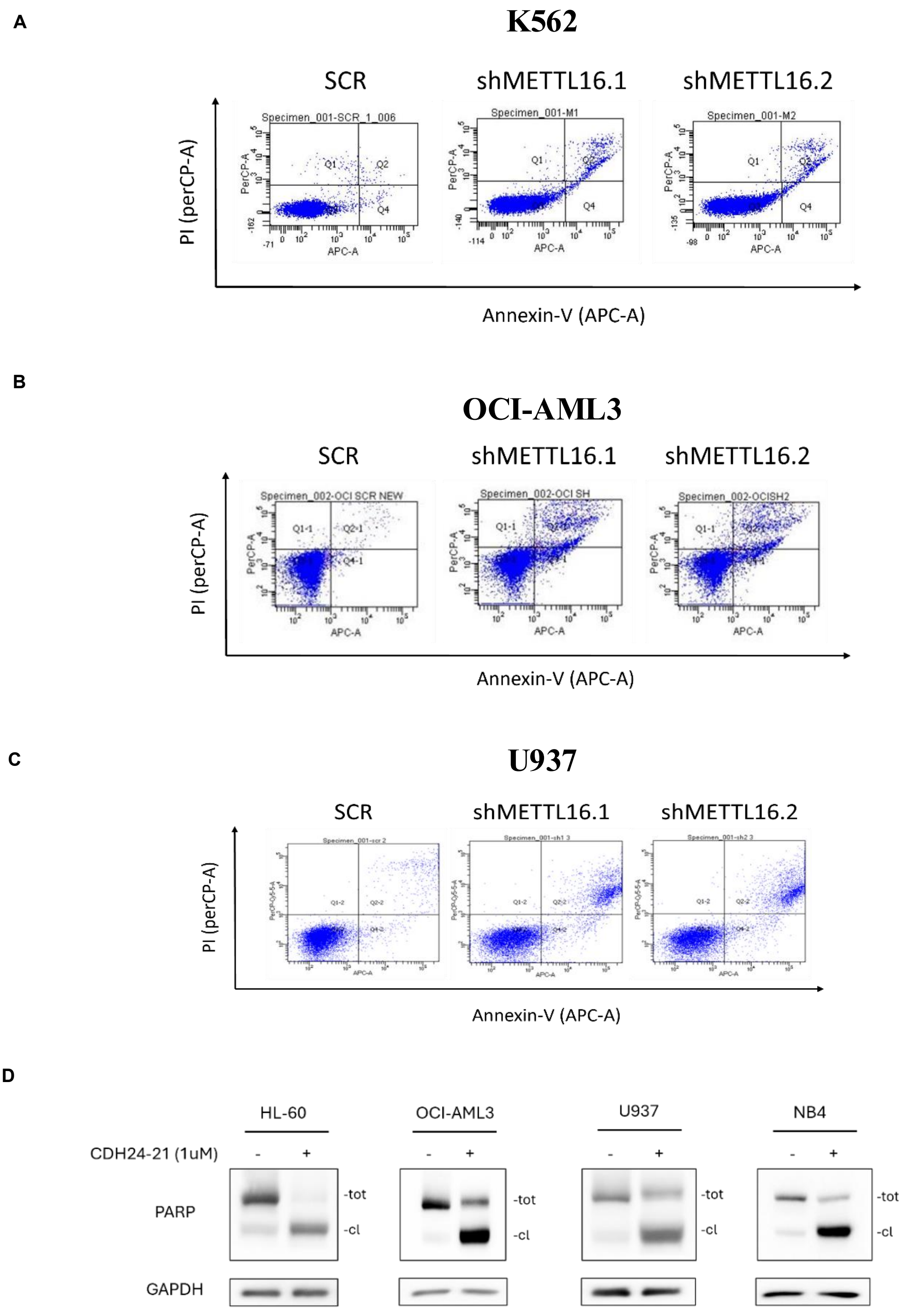

Figure S4. shMETTL16 induces apoptosis in AML cells (A-C). Annexin V/PI analysis showing apoptosis induction following SCR, shMETTl16.1 and shMETTl16.2 transduction in (A) K562, (B) OCI-AML3, and (C) U937 cell lines (n=3). **(D)** WB analysis of PARP protein levels in HL-60, OCI-AML3, U937 and NB4 cell lines following 72 h treatment with CHD24-21 at indicated concentration (n=3).

Supplementary figure 5

A

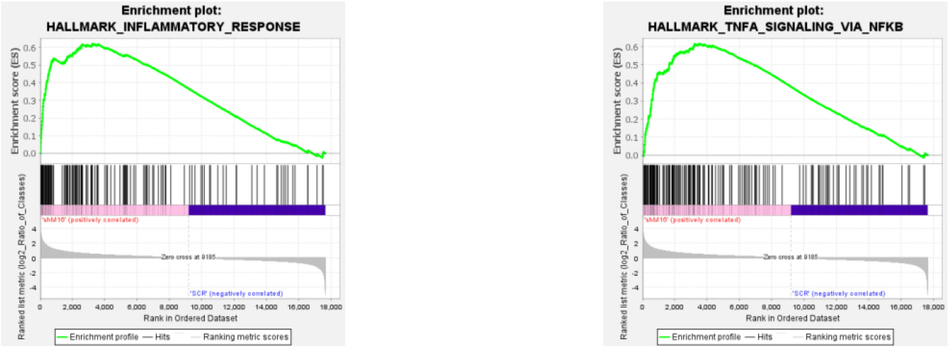

B

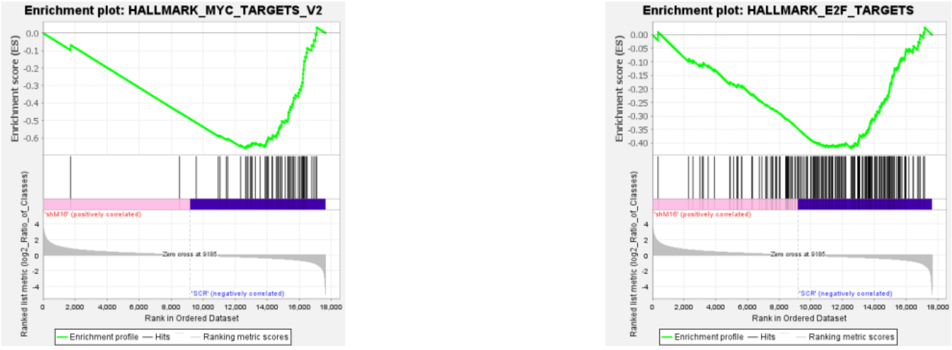

Figure S5. Top hallmark gene sets enriched in SCR and shMETTL16. (A and B) GSEA analysis showing the top hallmark gene sets in (A) shMETTL16- (*P*< 0.01 and FDR<0.01) and (B) SCR- transduced K562 cells (*P*< 0.01 and FDR<0.01).

Supplementary figure 6

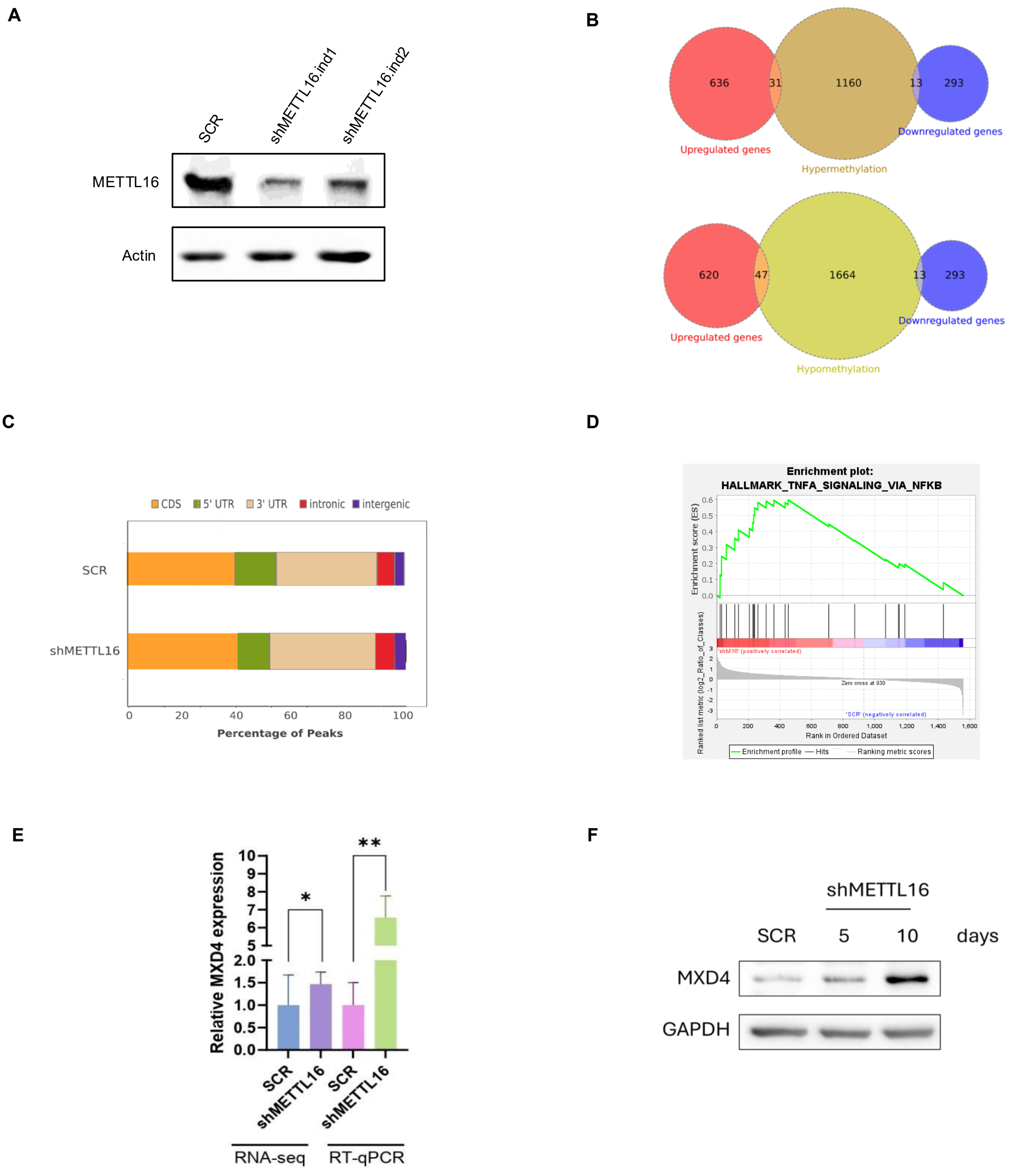

Figure S6. Effect of METTL16 KD on m^{6}A-epitranscriptome in K562 cells. (A) WB analysis of METTL16 protein levels in SCR, shMETTL16.1 and shMETTL16.2 inducible cells following 4 days of puromycin selection and treated with Doxycycline (1ug/mL) for 72h. **(B)** Venn diagram illustrating the overlap between MeRIP-seq and bulk RNA-seq data. In total, 1,724 genes were hypomethylated (lost m^{6}A), and 1,204 genes were hypermethylated (gained m^{6}A) following METTL16 depletion (|Log2FC≥1|; MeRIP-seq $p < 0.01$; RNA-seq adjusted $P < 0.05$).**(C)** meRIP analysis of m6A distribution along mRNA transcripts in SCR and shMETTL16 samples. **(D)** GSEA analysis of meRIP-seq showing the top hallmark gene sets of hypomethylated transcripts in shMETTL16-transduced cells compared to the SCR ($P< 0.05$). **(E)** Expression analysis of MXD4 in SCR and METTL16-depleted cells performed by RNA-seq and RT-qPCR experiments (*$p<0.05$; **$p \leq 0.01$) (n=3). **(F)** WB analysis of MXD4 protein levels in SCR and shMETTL16 at the indicated time points (n=3). Student's t-test was used to calculate statistical significance in E.

Supplementary figure 7

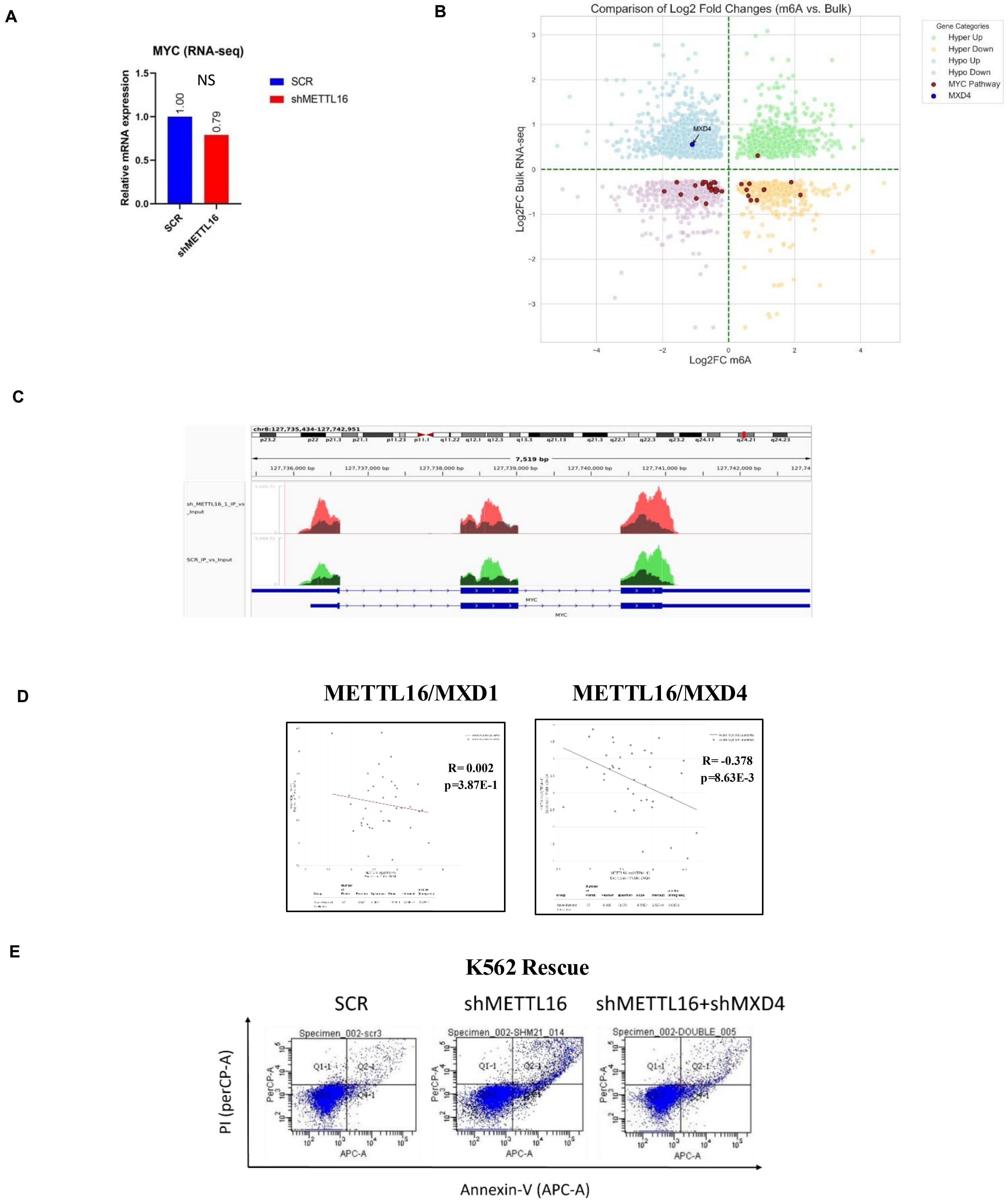

Figure S7. METTL16 depletion effects on MYC expression and rescue experiments. (A) Expression of MYC in SCR and shMETTL16 cells (from RNA-seq, 5 days post puromycin selection, data are not significant). **(B)** Scatter plot depicting the log2 fold changes in methylation levels of gene transcripts (x-axis) versus the log2 fold changes in gene expression (y-axis). MXD4 and MYC target genes are highlighted (P<0.05). **(C)** IGV screenshot showing no alteration of MYC transcript methylation in SCR and shMETTL16 K562 cells (from MeRIP-seq, data are not significant). **(D)** Expression correlations between METTL16/MXD1 and METTL16/MXD4 in AML cells (Dep Map; **P<0,01) **(E)** Annexin V/PI assay of SCR-, shMETTL16- and shMETTL16/shMXD4- transduced K562 cell line (n=3).

Supplementary figure 8

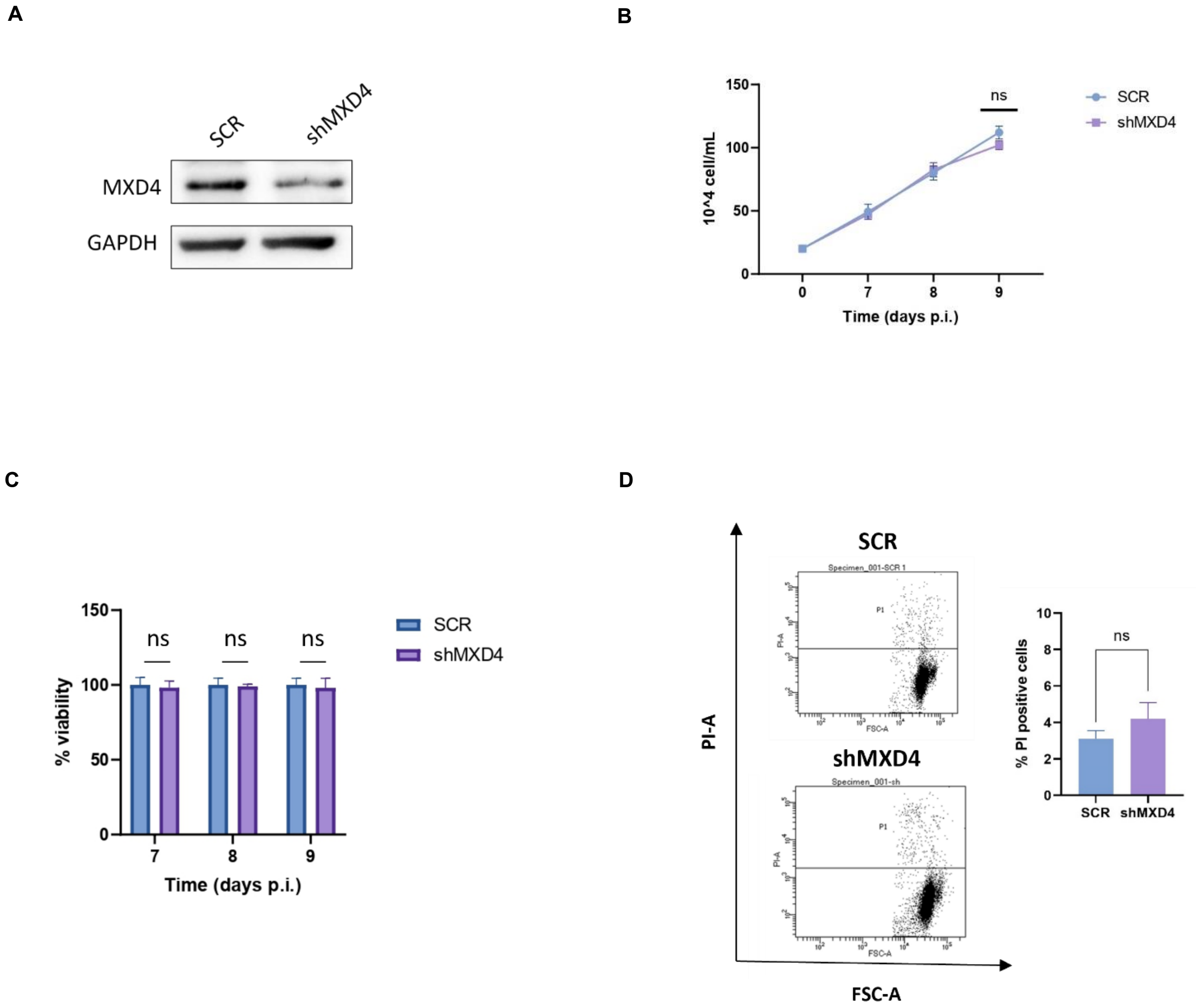

Figure S8 Depletion of MXD4 does not influence leukemic cell proliferation (A) WB analysis showing MXD4 protein levels in SCR-, and shMXD4-transduced K562 cells (n=3). **(B)** Proliferation assay of shMXD4- and SCR-transduced K562 cells (n=3). **(C)** CCK8 viability assay in shMXD4- and SCR-infected K562 (n=3). **(D)** PI analysis of MXD4-silenced K562 cells (n=3).
